# Phylogenetic and Molecular Evolutionary Analysis of Mitophagy Receptors under Hypoxic Conditions

**DOI:** 10.3389/fphys.2017.00539

**Published:** 2017-07-26

**Authors:** Xiaomei Wu, Fei-Hua Wu, Qianrong Wu, Shu Zhang, Suping Chen, Matthew Sima

**Affiliations:** ^1^College of Life and Environmental Sciences, Hangzhou Normal University Hangzhou, China; ^2^Department of Biology, Duke University Durham, NC, United States; ^3^College of Life Sciences, Zhejiang University Hangzhou, China

**Keywords:** mitophagy receptor, hypoxia, oxygen concentrations, FUNDC1, BNIP3, NIX, molecular evolution, land vertebrates

## Abstract

As animals evolved to use oxygen as the main strategy to produce ATP through the process of mitochondrial oxidative phosphorylation, the ability to adapt to fluctuating oxygen concentrations is a crucial component of evolutionary pressure. Three mitophagy receptors, FUNDC1, BNIP3 and NIX, induce the removal of dysfunctional mitochondria (mitophagy) under prolonged hypoxic conditions in mammalian cells, to maintain oxygen homeostasis and prevent cell death. However, the evolutionary origins and structure-function relationships of these receptors remain poorly understood. Here, we found that FUN14 domain-containing proteins are present in archaeal, bacterial and eukaryotic genomes, while the family of BNIP3 domain-containing proteins evolved from early animals. We investigated conservation patterns of the critical amino acid residues of the human mitophagy receptors. These residues are involved in receptor regulation, mainly through phosphorylation, and in interaction with LC3 on the phagophore. Whereas FUNDC1 may be able to bind to LC3 under the control of post-translational regulations during the early evolution of vertebrates, BINP3 and NIX had already gained the ability for LC3 binding in early invertebrates. Moreover, FUNDC1 and BNIP3 each lack a layer of phosphorylation regulation in fishes that is conserved in land vertebrates. Molecular evolutionary analysis revealed that BNIP3 and NIX, as the targets of oxygen sensing HIF-1α, showed higher rates of substitution in fishes than in mammals. Conversely, FUNDC1 and its regulator MARCH5 showed higher rates of substitution in mammals. Thus, we postulate that the structural traces of mitophagy receptors in land vertebrates and fishes may reflect the process of vertebrate transition from water onto land, during which the changes in atmospheric oxygen concentrations acted as a selection force in vertebrate evolution. In conclusion, our study, combined with previous experimental results, shows that hypoxia-induced mitophagy regulated by FUDNC1/MARCH5 might use a different mechanism from the HIF-1α-dependent mitophagy regulated by BNIP3/NIX.

## Introduction

The survival of eukaryotic cells requires energy to perform different functions. Mitochondria, known as the powerhouse of the cell, play critical roles in the generation of all necessary biological energy of the cell through the process of mitochondrial oxidative phosphorylation. While mitochondrial oxidative phosphorylation is a highly efficient way of synthesizing ATP in aerobic organisms, in comparison to anaerobic glycolysis, the mitochondrion is also a primary source of reactive oxygen species (ROS) as a result of electrons prematurely reacting with oxygen (Zhang et al., 2008). Transient and low levels of ROS serve as signaling molecules to maintain normal physiological processes. However, prolonged elevations of ROS can induce oxidative stress, which causes damage to mitochondria themselves, and the damaged mitochondria produce more ROS in a vicious circle, ultimately leading to mitochondrial DNA damage (Kurihara et al., 2012). If the elevated production of ROS is propagated throughout the cell, it could cause damage to DNA, lipids and proteins leading to cell dysfunction or death (Schieber and Chandel, 2014; Zorov et al., 2014). Therefore, oxygen concentrations must be tightly controlled to maintain energy and redox homeostasis. During oxygen deprivation (also called hypoxia), mitophagy serves as an adaptive metabolic response to prevent excess ROS production through selective elimination of damaged and dysfunctional mitochondria, which prevents cell death (Zhang et al., 2008). Defects in mitophagy can result in the accumulation of dysfunctional mitochondria and increased ROS production in cells, which has been linked to numerous human diseases, including cancers, age-associated neurodegenerative diseases (such as Parkinson disease), muscle atrophy, aging, metabolic disorders and heart failure (Chan, 2006; Palikaras and Tavernarakis, 2012; Springer and Macleod, 2016).

Bcl-2/adenovirus E18 19-kDa-interacting protein 3 (BNIP3) and its homolog NIX (also known as BNIP3L) are mitochondrial outer membrane proteins. They have been reported to be related to the Bcl-2 homology 3 (BH3) domain-only family, which induces programmed cell death, as previously reviewed (Zhang and Ney, 2009; Ney, 2015). The BH3 domain of NIX has signatures of binding and exchanging membrane lipids (Degli Esposti, 2002). Apart from their roles as pro-apoptotic proteins in cell death, BNIP3, and NIX function as mitophagy receptors, which mediate mitophagy during hypoxia (Zhang et al., 2008; Novak et al., 2010). Moreover, NIX is required for the selective elimination of healthy mitochondria during red blood cell differentiation and maturation (Novak et al., 2010). Under hypoxia conditions, the expression levels of BNIP3 and NIX are greatly increased through transcriptional regulation by hypoxia-inducible factor 1α (HIF-1α) (Zhang et al., 2008; Bellot et al., 2009). Hypoxia-induced BNIP3 and NIX initiate mitophagy, which has been attributed to an effect on the complex of Bcl-2 and Beclin-1 (Bellot et al., 2009). Mitophagy receptors BNIP3 and NIX directly interact with microtubule-associated protein 1 light chain 3 (LC3, also known as Atg8) and GABARAP at the phagophore membrane via a tetrapeptide sequence (WVEL) in the cytoplasmic region, and promote the sequestration of mitochondria in an autophagosome. The tetrapeptide sequence belongs to a classical LC3 interaction region (LIR) with a Y/W/F-x-x-L/I/V motif, which is also known to be present in several other Atg8- or LC3-binding proteins necessary for selective autophagy (Kanki et al., 2009; Okamoto et al., 2009). Furthermore, almost all of NIX's activity localizes to a novel short linear motif (SLiM) with 11 amino acid residues in its cytoplasmic region (Zhang et al., 2012).

A recently reported mitophagy receptor, FUN14 domain-containing protein 1 (FUNDC1), plays an essential and specific role in hypoxia-induced mitophagy in mammalian cells (Liu et al., 2012). While BNIP3 and NIX are transcriptionally up-regulated by HIF-1α in response to hypoxia, FUNDC1 is down-regulated during hypoxia-induced mitophagy. Like BNIP3 and NIX, FUNDC1 in human interacts with LC3 directly through its typical LIR motif Y(18)-x-x-L(21). FUNDC1 is regulated through ubiquitylation and degradation by MARCH5 (Chen et al., 2017) and through reversible phosphorylation at several key sites (Chen et al., 2014; Wu et al., 2014). The molecular details of the precise working mechanism of FUNDC1 in mammalian cells was proposed by structural and biochemical studies (Kuang et al., 2016; Lv et al., 2017). Moreover, two studies (Chen et al., 2016; Wu et al., 2016) bring FUNDC1 into an interface between mitochondria and the endoplasmic reticulum, where FUNDC1 controls mitochondrial dynamics and mitophagy by coordinating the interactions with OPA1 and DRP1. Currently it is unknown which of BNIP3, NIX and FUNDC1 plays a more important role in inducing mitophagy during hypoxia, but dephosphorylated FUNDC1 shows much stronger binding than NIX with LC3 (Novak et al., 2010).

During metazoan evolution, vertebrates migrated from water to land in the Late Devonian period, approximately 367 million years ago (Pyron, 2011), and this was an important step in the evolutionary history of modern land vertebrates. This transition allowed animals to escape competition pressure in the water and acquire more oxygen on land. Water contains a much lower concentration of oxygen than the air in the atmosphere. During the ~550 million years of animal evolution, oxygen levels in the atmosphere have varied between 15 and 30% (Berner et al., 2007). These changing oxygen levels have affected metazoan evolution. HIFα (HIF-1α, HIF-2α, and HIF-3α) are primary regulators of the adaptive transcriptional response to hypoxia. The HIFα oxygen-sensing pathway is highly conserved; however, the complexity (in the form of numbers of HIFα duplicates) of HIFα increased during the periods when atmospheric oxygen levels were rising (Taylor and McElwain, 2010; Rytkonen et al., 2011). Moreover, the rate and mode of molecular evolution of HIF-1α are different in water-breathing fishes and air-breathing mammals, which has been linked to the different oxygen tensions in water and on land (Rytkonen et al., 2008). Animals evolved to utilize oxygen as the main strategy to produce metabolic energy in the form of ATP. Thus, how to adapt to fluctuating oxygen conditions is a key component of evolutionary selection in animals (Taylor and McElwain, 2010).

The three mitophagy receptors, BNIP3, NIX, and FUNDC1, induce mitophagy under prolonged hypoxia in mammalian cells for the maintenance of redox homeostasis and the survival of hypoxic cells. However, their evolutionary origins and scenarios remain largely elusive. In this study, we aimed to present the first comprehensive phylogenetic and molecular evolutionary analysis of the three mitophagy receptor families in response to oxygen deprivation. FUNDC1 contains a conserved FUN14 domain and both BNIP3 and NIX contain a BNIP3 domain. First, systematic homolog searches revealed that the FUN14 domain-containing protein family is present in nearly all three domains of living organisms (eukaryotes, archaea, and bacteria), whereas the BNIP3 domain-containing protein family is present only in animals. Phylogenetic analysis showed that both families contain two paralogous subfamilies in vertebrates, namely, FUNDC1/FUDNC2 and BNIP3/NIX, both of which probably resulted from whole-genome duplication (WGD) in the vertebrate ancestor. Then, evolutionary constraints on different gene copies after WGDs were investigated. To understand evolutionary insights into the structure-function relationships of FUNDC1, BNIP3 and NIX, we examined conservation patterns of the classical LIR motif, the SLiM (essential for the activity of NIX) and the key phosphorylation residues across metazoan genomes. We also present an evolutionary profile of the molecular components in the mitophagy pathway across eukaryotic genomes. Finally, we investigated patterns in the molecular evolution of the three mitophagy receptors under hypoxia and their interacting proteins in water-breathing teleost fishes and air-breathing mammals. Reverse substitution patterns between FUNDC1 and BNIP3/NIX were revealed. This study allows us to explore the origins of mitophagy receptors in response to hypoxic stress, and to deduce the potential selection forces on the evolution of these receptors.

## Materials and methods

### Data sources and identification of orthologs

A list of fully sequenced genomes of eukaryotes (253 species), bacteria (2480 species) and archaea (163 species) was derived from the Database of KEGG Organisms (http://www.genome.jp/kegg/catalog/org_list.html). Protein sequences of all species and mRNA sequences of animal genomes were obtained from RefSeq (ftp://ftp.ncbi.nih.gov/refseq/release/). The genome data of Ctenophore *Mnemiopsis leidyi*, which evolved in early metazoans, was from the Mnemiopsis Genome Project Portal (http://research.nhgri.nih.gov/mnemiopsis/).

FUNDC1 contains a conserved FUN14 domain. BNIP3 and NIX contain a BNIP3 domain. DRP1 and OPA1, both of which interact with FUNDC1, belong to the “dynamin superfamily”. Proteins containing a FUN14 (PF04930), BNIP3 (PF06553) or dynamin (PF00350) domain in fully sequenced eukaryotic and prokaryotic genomes were detected using three steps: (1) Command hmmsearch in the HMMER package v3.1b was first run by querying the Pfam-A PF04930, PF06553 or PF00350 Hidden Markov Model (HMM) against the protein sequences of all species. A sequence *E* ≤ 1e-2 and a conditional *E* ≤ 1e-2 for individual domains were adopted to identify significant protein matches; (2) To confirm the presence of a FUN14, BNIP3 or dynamin domain in the obtained proteins, these proteins were compared against the library of Pfam-A HMMs (http://ftp.ebi.ac.uk/pub/databases/Pfam/current_release/) using the Pfam_scan.pl script (http://ftp.ebi.ac.uk/pub/databases/Pfam/Tools/) (parameters: -e_seq 1e-3 -e_dom 1e-3). The sequences covering equal to or more than 50% of the query domain were kept; (3) To remove redundant protein sequences that were resulted from alternative splicing, CD-HIT v4.6 (Fu et al., 2012) was used (parameters: -c 0.95 -n 5 -g 1 -G 0 -aS 0.6 -d 0 -p 1).

DRP1 and OPA1 were identified according to the phylogenetic analysis of the dynamin superfamily. Eukaryotic orthologs of the other eight proteins (HIF-1α, MARCH5, CK2, SRC, PGAM5, ULK1, calnexin and LC3) related to BNIP3 and NIX and FUNDC1 were identified using a bidirectional BLASTP search. First, each of the eight human protein was considered a “query” and was initially searched against the eukaryotic proteome dataset via BLASTP (Altschul et al., 1997) at an *E* ≤ 1e-3. Then, the obtained hits were reversely aligned against the human proteome at an *E* ≤ 1e-3. The putative orthologs were identified if the reverse BLASTP best hit is the query human protein. Third, CD-HIT was used to remove redundant sequences. We did not use a bidirectional best BLASTP search because sometimes the putative ortholog does not rank first during the first round of BLASTP.

The amphibian lineage only contains FUNDC1, but lacks FUNDC2. Two amphibian genomes, *Xenopus laevis* (Xla) and *Xenopus tropicalis* (Xtr), were fully sequenced and their genomic information is stored in Xenbase (http://www.xenbase.org/). To check whether there is any error in sequence annotation that leads to the lack of FUNDC2 in amphibians, we aligned the protein sequence of FUNDC2 in zebrafish *D. rerio* against the genomic sequences of *X. laevis* and *X. tropicalis* using TBLASTN, but failed to find any indication of FUNDC2 homologs in the amphibian genomes. Choanoflagellate *Monosiga brevicollis* is the closest living relative of metazoans. Six proteins (HIF-1α, BNIP3, MARCH5, SRC, PGAM5, and ULK1) have metazoan homologs, but lack homologs in *M. brevicollis*. To confirm the absence of these proteins in *M. brevicollis*, we aligned these six proteins in animals against the *M. brevicollis* genomic sequences using TBLASTN at the JGI Genome Portal (http://genome.jgi.doe.gov/). However, we found no indication of a homolog in *M. brevicollis*. The full datasets of FUN14 domain-containing proteins (297 sequences from 224 eukaryotic and prokaryotic genomes), BNIP3 domain-containing proteins (142 sequences from 81 metazoan genomes) and MARCH5 proteins (185 sequences from 128 eukaryotic genomes) are listed in Supplementary Table 1 and their protein sequences are available in Supplementary Data 1.

### Phylogenetic analysis

A phylogenetic tree of dynamin domain-containing proteins in eukaryotes was reconstructed to classify DRP1 and OPA1 from the dynamin superfamily. Sequences that were too short (shorter than 300 aa) or too long (longer than 1,500 aa) were removed from phylogenetic analysis. A total of 2352 eukaryotic dynamin domain-containing proteins were obtained. Multiple sequence alignments of these proteins were performed using MUSCLE v3.8 (Edgar, 2004), with the maximum number of iterations set at 2. The phylogeny was created using FastTree v2.1.7 (Price et al., 2009) with its default parameters. FastTree implements an ultrafast and fairly accurate approximate maximum-likelihood method.

Multiple sequence alignments of the other protein sequences were performed using MAFFT v7.05 (Katoh and Standley, 2013) with the L-INS-i method. The phylogenetic relationship of 297 FUN14 domain-containing proteins in all three domains of organisms was reconstructed using two methods, namely, FastTree and a neighbor-joining method in MEGA6 (Tamura et al., 2013). In MEGA6, bootstrap was performed with 1,000 replicates and gaps were treated as pairwise deletion.

To further analyze the phylogenetic relationship of gene families within the metazoan lineage, we sampled animal species that represent a wide variety of metazoan lineages (Table 1). Three subsets of 53 FUN14 domain-containing proteins, 56 BNIP3 domain-containing proteins, and 44 MARCH5 homologs were obtained (detailed information is available in Supplementary Table 1 and their FASTA sequences can be found in Supplementary Data 1). Protozoan and/or fungal homologs were selected as outgroups to root the trees of FUN14 and MARCH5. DDX54 in animals was set as outgroups of the BNIP3 trees. For each protein dataset, three phylogenetic trees (multiple sequence alignment files in FASTA format and tree files in Newick format are available in Supplementary Data 2) were created. Neighbor-joining, Bayesian and maximum-likelihood trees were estimated using MEGA6, MrBayes v3.2.3 (Ronquist et al., 2012) and PhyML v3.1 (Guindon et al., 2010), respectively. In MEGA6, bootstrap was performed with 1000 replicates and gaps were treated as pairwise deletion. The Akaike Information Criterion implemented in ProtTest v3.4 (Darriba et al., 2011) determined the best-fit models of evolution as LG+I+G for FUN14 trees, JTT+G for BNIP3 trees and LG+I+G+F for MARCH5 trees. A maximum-likelihood tree was estimated with 100 bootstrap replicates. Bayesian analysis was performed on two parallel runs, each with the distribution posterior probability of the generated trees estimated using Markov Chains Monte Carlo with four chains (1 cold, 3 heated). Convergence between the two parallel runs was ascertained by examining the average standard deviation of their split frequencies as <0.01. A total of 3,000,000 generations and 1,000 subsampling frequencies were set. Consensus topology and posterior probability values were computed from saved trees after discarding 25% of generations as burn-in. Phylogenetic trees were visualized in FigTree (http://tree.bio.ed.ac.uk/software/figtree/).

**Table 1:** Representative metazoan species used in phylogenetic and molecular evolutionary analysis.

**Lineage**	**Species**	**Abbr.^a^**	**TaxonID^b^**
Mammals	*Homo sapiens* (human)	Hsa	9606
Mammals	*Pongo abelii* (Sumatran orangutan)	Pon	9601
Mammals	*Mus musculus* (mouse)	Mmu	10090
Mammals	*Canis familiaris* (dog)	Cfa	9615
Mammals	*Bos taurus* (cow)	Bta	9913
Mammals	*Sus scrofa* (pig)	Ssc	9823
Mammals	*Monodelphis domestica* (opossum)	Mdo	13616
Mammals	*Ornithorhynchus anatinus* (platypus)	Oaa	9258
Birds	*Gallus gallus* (chicken)	Gga	9031
Birds	*Taeniopygia guttata* (zebra finch)	Tgu	59729
Reptiles	*Alligator sinensis* (Chinese alligator)	Asn	38654
Reptiles	*Pelodiscus sinensis* (Chinese soft-shelled turtle)	Pss	13735
Amphibians	*Xenopus laevis* (African clawed frog)	Xla	8355
Amphibians	*Xenopus tropicalis* (western clawed frog)	Xtr	8364
Lobe-finned fishes	*Latimeria chalumnae* (coelocanth)	Lcm	7897
Teleost fishes	*Danio rerio* (zebrafish)	Dre	7955
Ray-finned fishes	*Lepisosteus oculatus*	Locu	7918
Lancelets	*Branchiostoma floridae* (Florida lancelet)	Bfo	7739
Ascidians	*Ciona intestinalis* (sea squirt)	Cin	7719
Echinoderms	*Strongylocentrotus purpuratus* (purple sea urchin)	Spu	7668
Arthropods	*Drosophila melanogaster* (fruit fly)	Dme	7227
Arthropods	*Drosophila willistoni*	Dwi	7260
Arthropods	*Anopheles gambiae* (mosquito)	Aga	180454
Arthropods	*Tribolium castaneum* (red flour beetle)	Tca	7070
Molluscs	*Octopus bimaculoides* (cephalopod)	Obi	37653
Nematodes	*Caenorhabditis elegans*	Cel	6239
Nematodes	*Caenorhabditis briggsae*	Cbr	6238
Cnidarians	*Nematostella vectensis* (sea anemone)	Nve	45351
Placozoans	*Trichoplax adhaerens*	Tad	10228
Ctenophores	*Mnemiopsis leidyi*	Mld	27923

a *Three- or four-letter abbreviation of species names*.

b *Taxon id obtained from NCBI*.

### Selective pressure analysis

Estimation of the relative rate of non-synonymous and synonymous substitutions (ω = dN/dS) during the evolution of FUNDC1 and FUNDC2 as well as BNIP3 and NIX in vertebrates was performed using the CODEML program in the PAML package v4.7 (Yang, 1997). dN means the number of synonymous substitutions per synonymous site and dS means the number of non-synonymous substitutions per non-synonymous site. The ω ratio is a measure of natural selection acting on a protein sequence. Values of ω < 1, ω = 1 and ω > 1 indicate negative or purifying selection (acting against change), neutral (i.e., no) selection and positive Darwinian selection (driving change), respectively. Codon multiple alignments of mRNA sequences were created from the protein alignments using PAL2NAL (Suyama et al., 2006). Codon alignment and tree topology were then input to CODEML. Three different classes of codon substitution-based evolutionary models, namely, branch model (Yang, 1998), clade model and branch-site model (Yang and Nielsen, 2002), were adopted. These models perform maximum-likelihood estimates of ω ratios and produce a log-likelihood (ln *L*) value to each examined alignment and phylogeny. (1) To assess whether the “foreground” lineage evolved under a different selection relative to the rest of the phylogeny (“background” lineage), branch models were used. A likelihood ratio test (LRT) was run to compare a two-ratio branch model, which allows the ω ratio to vary among the foreground and background lineages, against a one-ratio null model, which constrains all branches in the phylogeny to the same ω. Then, a better fitting model from the comparison was evaluated if the *P*-value for fitting the examined dataset to the alternative model was ≤0.05. (2) Clade model C was performed to test for divergent selection pressures acting on one class of sites between two subfamilies following gene duplication. LRT was run to evaluate the significance of clade model C against the site-specific null model M2a_ref (Weadick and Chang, 2012). (3) To detect whether positive selection had acted at some amino acid sites within the foreground lineage, branch-site test 1 (Zhang et al., 2005) was used. Branch-site model A, featuring an extra subset of sites under positive selection with ω > 1 in the foreground lineages, was compared with site model M1a (“nearly neutral”) as the null hypothesis. Significance of the test can be caused either by relaxed selective constraint on the foreground lineage (e.g., presence of sites with ω_2_ = 1 on the foreground and ω_2_ < 1 on the background) or by positive selection along the foreground lineage. In this study, relaxed selection was concluded to be exerted somewhere along the sequences in FUNDC2. Clade model C and branch-site model A were performed multiple times using different initial values of ω (0.001, 0.01, 0.1, 0.25, 0.5, 0.75, 1–5, and 10 for Clade model C, and 1.25, 1.5, 1.75, 2, 2.5, 3, 3.5, 4–7, and 10 for model A) to find the overall optimum ln *L*.

### Functional divergence analysis

Functional divergence of two subfamilies after gene duplication was inferred by type I (Gu, 1999) and type II (Gu, 2006) divergence analysis using DIVERGE v3 (Gu et al., 2013) with 500 bootstrap replications. Type I represents heterogeneous evolutionary rates between duplicated genes. Type II represents amino acid patterns that are highly conserved within both subfamilies but have radical shifts of biochemical properties between duplicates. Coefficients of functional divergence and posterior probabilities of amino acid sites to be responsible for functional divergence were estimated. A *P*-value was calculated by comparing the coefficient (LRT Theta) of functional divergence with a chi-square distribution (one degree of freedom). Then, a suitable cutoff value of the posterior probability scores between two subfamilies was estimated to choose the amino acid sites reflecting functional divergence with a score larger than the cutoff value. Posterior probability values were sorted in descending order and then the highest scoring amino acid residues were consecutively removed from the alignment until the *P*-value of the coefficient became insignificant (>0.05). In this case, the remaining highest posterior probability was selected as a cutoff value. Three functional divergent amino acid sites of FUNDC1 and FUNDC2 were chosen with posterior probabilities larger than 0.811.

### Analysis of substitution patterns in different vertebrate clades

Two approaches were used to investigate patterns of substitution in different clades of vertebrates (teleost fishes, mammals, and land vertebrates). First, SNAP at https://www.hiv.lanl.gov/content/sequence/SNAP/SNAP.html (Korber, 2000) was used. For each of the mitophagy receptors and their interacting proteins, codon alignment sequences in each vertebrate clade were input into SNAP. dS and dN values were then calculated for each pair of sequences, and the average dS, dN and ω ratio (=dN/dS) were used in this study. For FUNDC1 and MARCH5, the dS and dN values were also computed based on each domain partition. Second, two-ratio models compared with the one-ratio model in CODEML were used to test whether the ω ratio for the clade of interest is different from other vertebrate clades. The models were tested on the phylogenies of FUNDC1, MARCH5, BNIP3 and NIX in vertebrates. For each gene, Benjamini-Hochberg multiple correction (Benjamini and Hochberg, 1995) was used to adjust the significance level.

### Syntenic cluster analysis

The Ohnologs database is a repository of paralogous genes retained from WGDs (i.e., ohnologs) in the vertebrate genomes (Singh et al., 2015) (http://ohnologs.curie.fr/). The Synteny Database (Catchen et al., 2009) (http://syntenydb.uoregon.edu/synteny_db/) is an automated system to identify conserved syntenic regions produced from WGD. NIX and BNIP3 were housed in the Ohnologs database as an ohnolog pair with an intermediate confidence level. The Synteny Database also discovered paralogous syntenic clusters between the regions around NIX and BNIP3 in human chromosomes 8 and 10, respectively. The Ohnologs database identified FUNDC1 and FUNDC2 as strict (the highest confidence level) ohnologs, but we did not find any syntenic cluster between them in the Synteny Database because human FUNDC1 and FUNDC2 genes are on the same chromosome (X). To identify paralogous syntenic clusters between the regions around FUNDC1 and FUNDC2 in human genome, we modified the online pipeline of the Synteny Database to conduct syntenic cluster analysis. First, paralogous proteins in human were retrieved through BioMart at http://www.ensembl.org/biomart/martview/. Second, the paralogous groups were filtered through outgroup anchoring. Lancelet *Branchiostoma floridae* was used as an unduplicated outgroup. Each member of each paralogous group was checked to determine its top BLASTP hit (*E* < 1e-10) in the outgroup genome. If a group member has no BLASTP hit in the outgroup, the member is discarded from that group. If members of a group have top BLASTP hits to different proteins in the outgroup, the group is split into subgroups, each of which is anchored to the appropriate ortholog in the outgroup. Third, conserved syntenic clusters were detected between the two human genomic regions, which individually contain FUNDC1 and FUNDC2. This was accomplished through a manually sliding window analysis (Catchen et al., 2009) to check whether the neighboring genes of the FUN14-containing paralogs keep their relative positions and orders in genomic regions. Inverted regions of conserved synteny were also considered. Visualization of syntenic clusters was performed using the genoPlotR package (Guy et al., 2010) in R (R Core Team, 2014).

## Results

### Whole-genome duplication in vertebrates leads to FUNDC1 and FUNDC2

**Figure 1:**
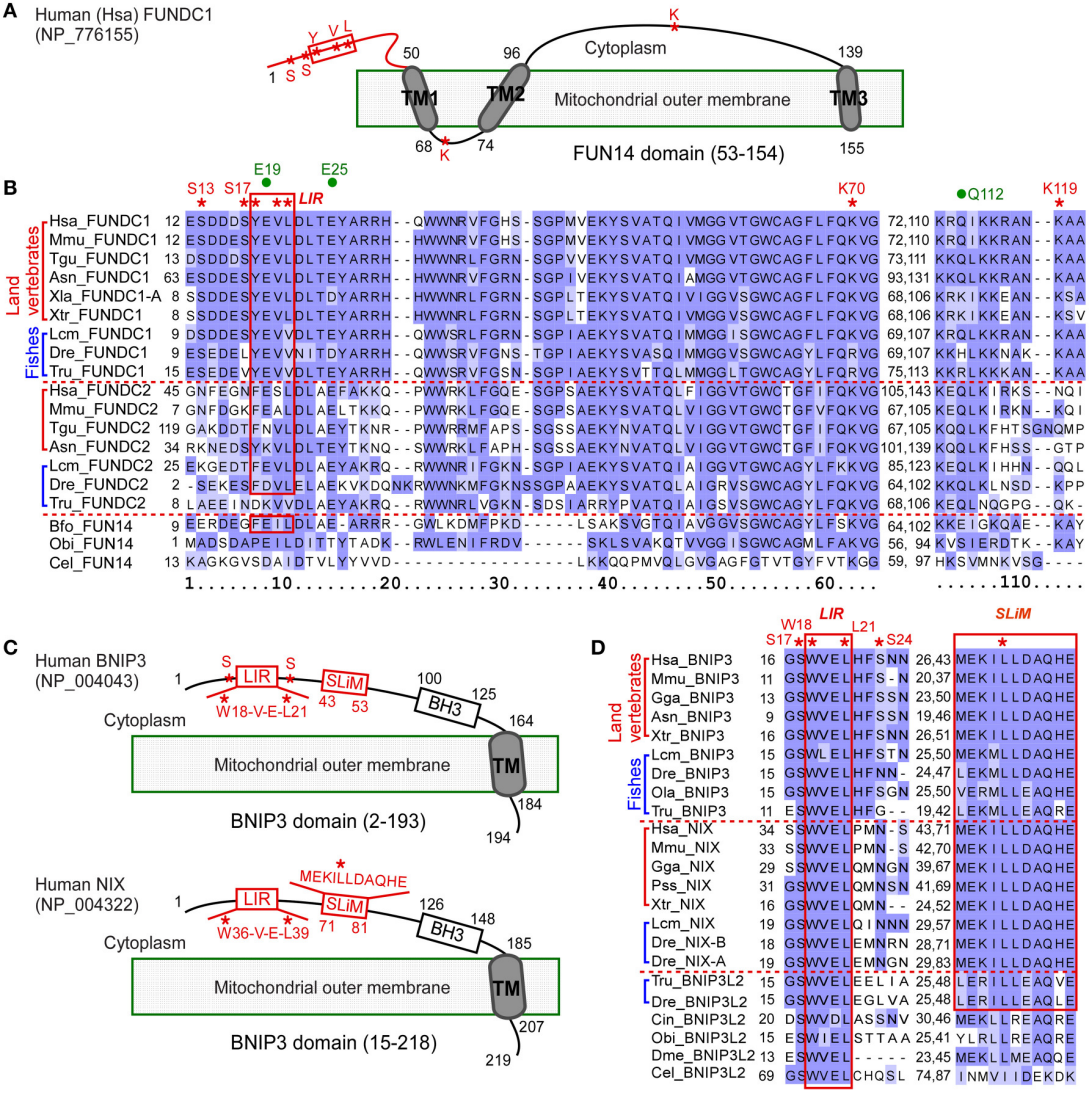
Multiple sequence alignment of proteins containing FUN14 or BNIP3 domain in animals. **(A)** Illustration representing the topology of *Homo sapiens* FUNDC1 (NP_776155), a mitochondrial outer membrane protein. Cytosolic region is at the top of the membrane. FUN14 domain (PF04930) covers all three transmembrane (TM) domains. Red box indicates LC3 binding region (LIR, Y/W/F-x-x-L/I/V). Red asterisks indicate key phosphorylation sites (S13, S17, and Y18), conserved sites in LIR, and key sites (K70, K119) for interacting with OPA1 and MARCH5. **(B)** Alignment of FUN14 domain-containing proteins in representative metazoan species of six land vertebrates, three fishes and three invertebrates. The LIR motif is enclosed within a red box. Red asterisks indicate biologically important residues as labeled in **(A)**. Three green dots indicate predicted functionally divergent sites (see Materials and Methods). **(C)** Topology of two other mitochondrial outer membrane proteins, BNIP3 (NP_004043) and its homolog NIX (NP_004322), in human. Both proteins contain a BNIP3 domain (PF06553), which covers a BH3 domain and a carboxyl-TM domain. They localize to the mitochondrial outer membrane through their C-terminal TM domains while their N termini are exposed to the cytosol. A short linear motif (SLiM) essential for NIX activity and an LIR motif are labeled with red boxes. The SLiM comprises a triplet of hydrophobic amino acid residues at its center and charged residues on each side of the triplet. Red asterisks indicate conserved sites in LIR, key phosphorylation sites (S17 and S24) in BNIP3, and the central leucine (Leu) residue in the hydrophobic triplet of SLiM in NIX. **(D)** Alignment of LIR motif and SLiM of BNIP3 and NIX in representative metazoan species. The LIR motif, W(18)-V-E-L(21), in human BNIP3 and its conserved sequences are enclosed within a red box. The SLiM are conserved in vertebrates and are enclosed within a red box. Red asterisks indicate key sites, as labeled in **(C)**. Ola indicates *O. latipes* and Tru indicates *T. rubripes*. Information on other species is listed in Table 1. The alignments in **(B,D)** were colored according to BLOSUM62 score in Jalview (Waterhouse et al., 2009).

**Figure 2:**
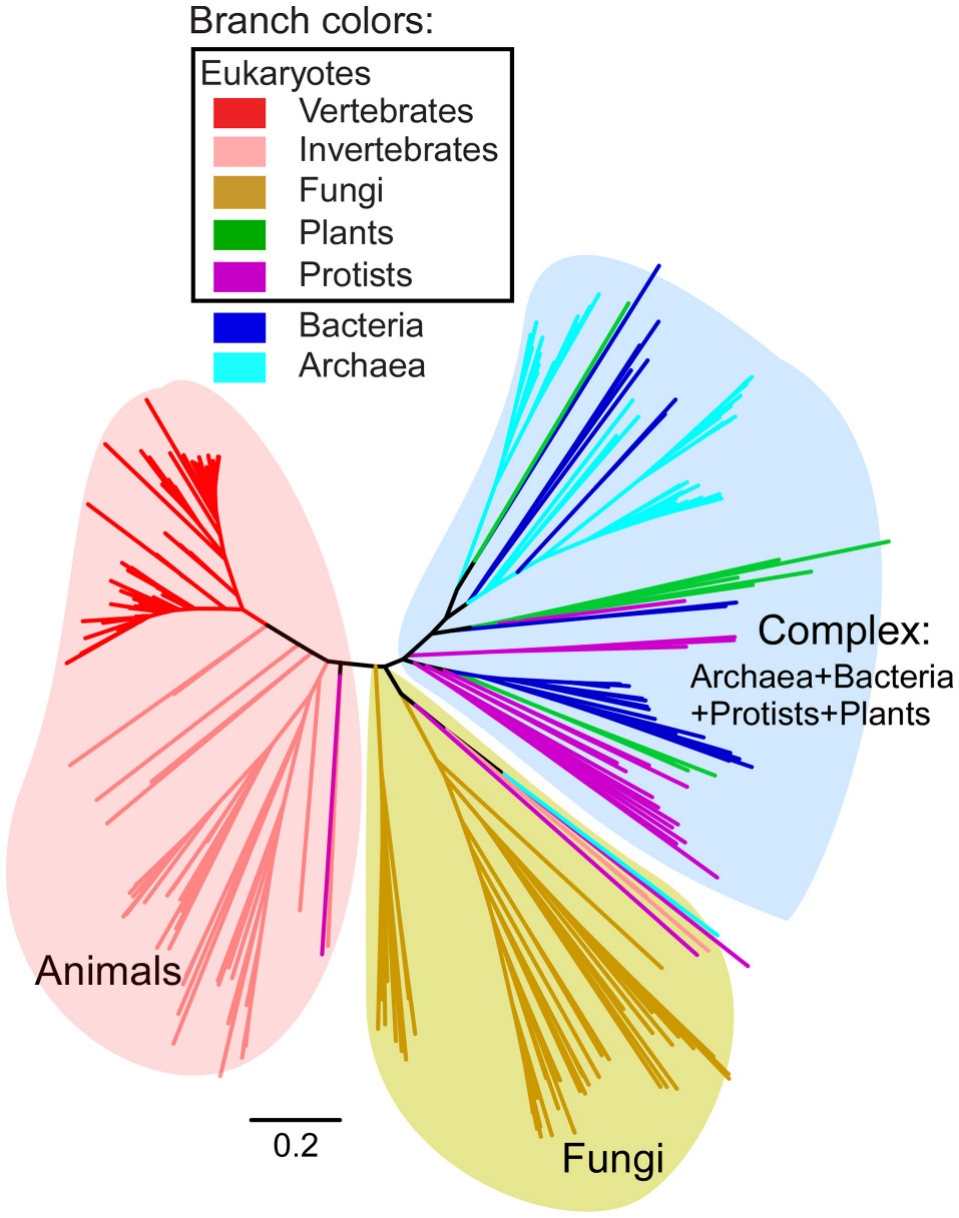
Phylogeny of full-length FUN14 domain-containing proteins in all three domains of life. HMM-based search for FUN14 domain was carried out in complete eukaryotic and prokaryotic genomes. A total of 297 FUN14 proteins from 145 eukaryotes and 79 bacteria and archaea were obtained. Multiple sequence alignment of the full-length proteins was performed using the MAFFT program (Katoh and Standley, 2013) with the L-INS-i method. The tree topology is from a neighbor-joining tree implemented in MEGA6 (Tamura et al., 2013) (see Materials and Methods). Branch lengths indicate the number of amino acid substitutions per site. Colored branches indicate different species lineages. Three major clades are identified, which are a metazoan clade, a fungal clade and a complex clade that includes FUN14 proteins from archaea, bacteria, protists and plants. Detailed information on the full dataset of FUN14 proteins is listed in Supplementary Table 1, and their sequences in FASTA format are available in Supplementary Data 1.

Human FUNDC1 consists of three transmembrane domains (TM1-TM3, amino acid residues 50–155 aa), a cytosol-exposed N-terminal region (1–49 aa) and a cytosol-exposed loop between TM2 and TM3 (Figure 1A). The conserved FUN14 domain (PF04930) covers the region (53–154 aa) between TM1 and TM3 of FUNDC1. A systematic and rigorous HMM-based search for the FUN14 domain was carried out in fully sequenced genomes of eukaryotes, bacteria and archaea (see Materials and Methods). The full dataset of FUN14 domain-containing proteins contains 297 sequences in 145 eukaryotic genomes and 79 prokaryotic genomes. Phylogenetic trees of the FUN14 domain-containing proteins (Figure 2, Supplementary Figure 1) divided these sequences into three clusters, which are a metazoan clade, a fungal clade and a complex clade that includes homologs from archaea, bacteria, protists and plants. FUN14 proteins are present in six bacterial phyla, which are Deinococcus-Thermus (19 proteins), Gammaproteobacteria (2), Epsilonproteobacteria (1), Deltaproteobacteria (2), Cyanobacteria (1), and Aquificae (2). Most of the archaeal FUN14 homologs are in Euryarchaeota (46 among 52 total genes) (Supplementary Table 1).

**Figure 3:**
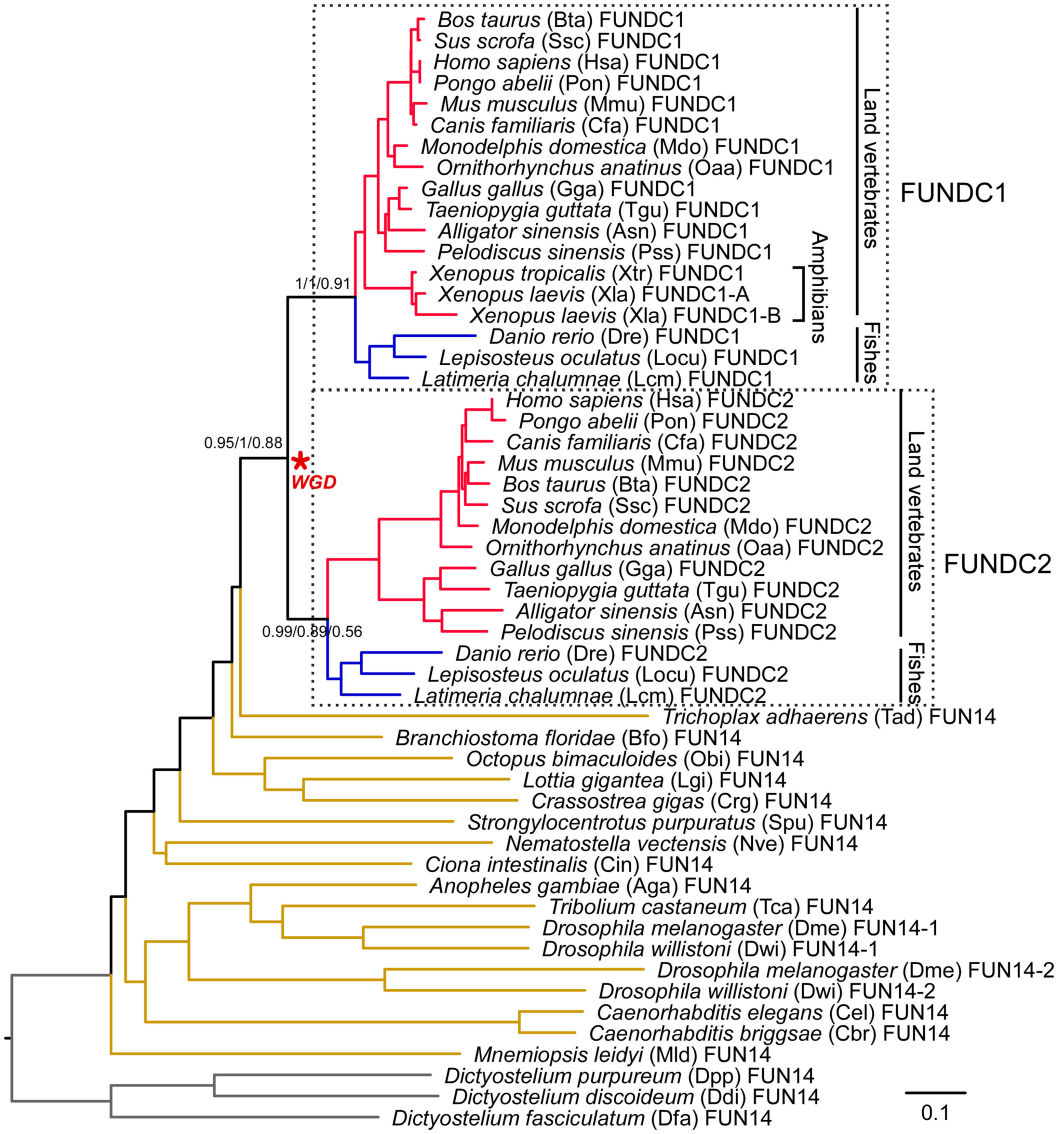
Phylogeny of FUN14 domain-containing proteins in animals. Thirty-three animal species, representing a wide variety of metazoan lineages, were sampled. Three protozoan orthologs from *Dictyostelium discoideum* (Ddi), *Dictyostelium fasciculatum* (Dfa), and *Dictyostelium purpureum* (Dpp) were selected as the outgroup to root the gene tree. A total of 53 FUN14 proteins were obtained in these species and were then aligned using MAFFT with the L-INS-i method. The tree topology is from a neighbor-joining tree. Statistical support values corresponding to bootstrap neighbor-joining, Bayesian posterior probability, and maximum-likelihood aLRT values are shown next to the corresponding nodes at relevant clades. Branch lengths are proportional to the evolutionary distances between nodes and are scaled with the number of amino acid substitutions per site. Each leaf node is depicted as a full species name, followed by a three- or four-letter abbreviation of the species name (some listed in Table 1) and a gene ID. The node defining the whole-genome duplication (WGD) leading to the subfamily of FUNDC1 and FUNDC2 in vertebrates is indicated with an asterisk. Branches in red, blue and brown denote land vertebrates, fishes and invertebrates, respectively. Branches in land vertebrates and fishes are also indicated with black lines. Two clades, clustering vertebrate FUNDC1, and FUNDC2 subfamilies, are enclosed within dashed boxes and are included in molecular evolutionary and functional divergence analyses. Detailed protein information is noted in Supplementary Table 1. Proteins sequences are available in Supplementary Data 1. Sequence alignments and three trees in Newick format are available in Supplementary Data 2.

To further analyze the phylogenetic relationship of FUN14 domain-containing proteins in the metazoan lineage, we created a subset of FUN14 proteins (FASTA sequences available in Supplementary Data 1) with 53 sequences in 33 animals representing various metazoan lineages (most species listed in Table 1) and three protists. Neighbor-joining, Bayesian and maximum-likelihood trees were constructed and rooted on protozoan homologs (Figure 3). Invertebrate genomes harbor only a single FUN14 domain-containing protein except the *Drosophila* lineage that contains two copies of the FUN14 genes, which may be the result of a gene duplication that occurred in the ancestor of the *Drosophila* genus, dating to ~40 million years ago (Clark et al., 2007). A comparison of the FUN14 domain-containing proteins in invertebrates and vertebrates exposes the emergence of two paralogs, namely, FUNDC1 and FUNDC2, in all vertebrates except amphibians (*X. laevis* and *X. tropicalis*). FUN14-containing paralogs in mammals localize to the same chromosome (X), whereas paralogs in non-mammalian vertebrates are distributed in different chromosomes, e.g., paralogs of zebrafish *D. rerio* are on chromosomes 9 and 21 (Supplementary Table 2). This suggests that genome rearrangement events have occurred in the regions around the FUN14-containing paralogs in the early evolution of mammals.

**Figure 4:**
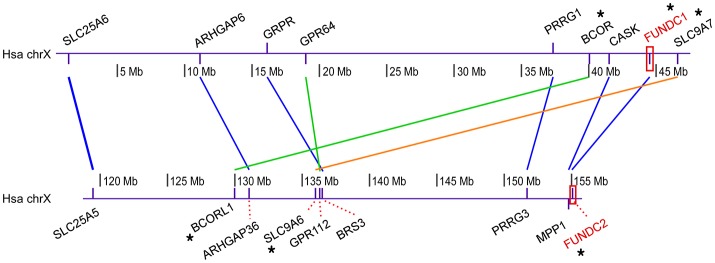
Syntenic cluster analysis among human FUNDC1 and FUNDC2. This analysis was carried out to evaluate the hypothesis that two FUN14 domain-containing proteins in vertebrates result from WGDs in the vertebrate ancestor, as opposed to simple tandem duplications. Human FUNDC1 and FUNDC2 (labeled in red) localize in two regions with a 100-Mb distance on the same chromosome (X) (Supplementary Table 2). The online pipeline of the Synteny Database (Catchen et al., 2009) was modified in this analysis. Paralogous proteins in the human genome were retrieved through BioMart and the paralogous groups were then filtered through outgroup anchoring. Lancelet *Branchiostoma floridae* was used as an unduplicated outgroup (see Materials and Methods). Finally, through a manually sliding window analysis, conserved syntenic clusters (the neighboring genes of FUNDC1 and FUNDC2 keep their relative positions and orders) were detected between the two human genomic regions, which individually contain FUNDC1 and FUNDC2. Colored lines between two paralogs denote different syntenic blocks. Nine gene pairs are shared in the paralogous syntenic clusters, in which three gene pairs are recorded in the Ohnologs database (Singh et al., 2015), which houses ohnologs—paralogous genes retained from WGD—in six vertebrate genomes.

The emergence of two FUN14-containing paralogs in all vertebrates is likely to be an outcome of two rounds of WGDs (shown with a red asterisk in Figure 3) that occurred in the genome of the vertebrate ancestor, before the divergence of ray-finned and lobe-finned fishes 450 million years ago (Braasch et al., 2016) (Supplementary Figure 2). To evaluate this hypothesis, as opposed to simple tandem duplications, a syntenic analysis was conducted on the human genome (see Materials and Methods). In this case, neighboring genes of duplicated genes have a tendency to retain their relative positions and orders on different chromosomes/genomic regions over evolutionary time; in other words, duplicated genes exist as part of conserved syntenic blocks. Indeed, paralogous syntenic clusters are observed between pairs of genomic regions containing FUNDC1 and FUNDC2, and nine conserved gene pairs are shared in the clusters (Figure 4). Furthermore, the Ohnologs database (Singh et al., 2015), which houses ohnologs—paralogous genes retained from WGD—in six vertebrate genomes, also tells us that three gene pairs in Figure 4, including FUNDC1 and FUNDC2, originate from WGD. Thus, the two FUN14-containing subfamilies in vertebrates result from WGD.

Gene duplication has long been thought as a primary source of material for the origin of evolutionary novelties, including new gene functions and expression patterns (Ohno, 1970). Changes in protein function may then yield different evolutionary constraints on gene copies after duplications (Taylor and Raes, 2004). To assess to what extent the natural selection has had on the FUN14 domain-containing proteins during vertebrate evolution and how the selective pressure is different among the gene copies after the gene duplication event, molecular evolutionary analysis was carried out at the coding region level. The non-synonymous-to-synonymous substitution ratio (ω = dN/dS) provides a measure of the selection pressure to which a gene is subject. ω > 1 indicates positive Darwinian selection (driving change). ω < 1 indicates purifying selection (acting against change). ω = 1 indicates neutral (i.e., no) selection. Different codon substitution-based evolutionary models in PAML were used to estimate ω in subfamilies of FUNDC1 and FUNDC2 (see Materials and Methods). The low ω ratio as 0.074 from one-ratio model, which assumes all lineages to have the same evolutionary rate, reflected strong purifying selection to act against mutations during the evolution of most of the FUN14 domain-containing proteins. The LRTs for the comparisons of two-ratio branch models, which assigned different ω ratios for individual subfamilies, with the one-ratio model indicated that the two subfamilies underwent asymmetrical evolution after gene duplication (*P* = 3.37E-07 in Supplementary Table 3). Furthermore, significant divergent selection pressures were presented at the sequence level in vertebrate FUNDC1 and FUNDC2 (*P* = 2.5E-09 in Table 2). The FUNDC2 clade was under weaker purifying selection (ω_Clade_FUNDC2_ = 0.248 in Table 2) than the FUNDC1 clade (ω_Clade_FUNDC1_ = 0.089), which was explained by relaxed selective pressure or relaxed functional importance instead of increased positive Darwinian selection at some sites (Supplementary Note 1). We also examined the phylogeny-based functional divergence of the FUN14 domain-containing protein family after duplication using DIVERGE (see Materials and Methods). Significant functional divergence type I, which represents heterogeneous evolutionary rates between duplicated genes, was detected in the lineages leading to vertebrate FUNDC1 and FUDNC2 (*P* = 1.86E-04 in Supplementary Table 4). The analysis also revealed that the two paralogous subfamilies experienced distinct functional constraints during their independent evolution after gene duplication. Three sites likely to be responsible for functional divergence of vertebrate FUNDC1 and FUDNC2 were also predicted (see Materials and Methods), and they are labeled with green dots in Figure 1B. For detailed information, please refer to Supplementary Note 1.

**Table 2:** Likelihood ratio tests for divergence in selective pressure among FUNDC1 and FUNDC2 in vertebrates by using clade model C.

**2 × ΔLn *L* (M2a_rel vs. Clade Model C)*^a^***	***P-*value**	**Site class**	**p^b^**	**ω_Clade_FUNDC1_^c^**	**ω_Clade_FUNDC2_^c^**
35.547	2.5E-09	0	0.57	0.018	0.018
		1	0	1	1
		2	0.43	0.089	0.248

a *ΔLn L = Ln L1 – Ln L0, where Ln L1 was the likelihood value of clade model C and Ln L0 (-5431.051) was the likelihood value of null model M2a_rel. Degrees of freedom = 2*.

b *Proportion of sites evolving under different site classes*.

c *Measure of natural selection acting on the FUNDC1 clade (ω_Clade_FUNDC1_) or FUNDC2 clade (ω_Clade_FUNDC2_)*.

### Evolutionary pattern of key residues of FUNDC1

FUNDC1 helps coordinate the signaling pathway mediating mitochondrial fission and fusion by interacting with MARCH5, OPA1, calnexin and DRP1 at the mitochondrion associated membrane, and helps coordinate the subsequent sequestration of the defective mitochondrion in an autophagosome by interacting with LC3. The cytosol-exposed N-terminal 50 residues of FUNDC1 in human is the determinant region for the interactions with LC3 (Kuang et al., 2016) and DRP1, the mitochondrial fission factor (Chen et al., 2016). In the N-terminal region, there is an LIR motif (Y18-E-V-L21) and three key phosphorylation sites (S13, S17, and Y18). Two sites, K70 and K119, bind to OPA1 and MARCH5, respectively, and are in the regions between two TM domains (Figures 1A,B). The binding of human FUNDC1 to LC3 relies principally on the four-residue LIR of FUNDC1 (Kuang et al., 2016). All of the four LIR residues of human FUNDC1 are identical in vertebrates, with the exception of in fish genomes, where the last aliphatic hydrophobic L residue is replaced by V, another aliphatic amino acid (Figure 1B). The LIR in human FUNDC1 belongs to a typical pattern of Y/W/F-x-x-L/I/V. The first aromatic residue (Y/W/F) and the fourth aliphatic hydrophobic residue (L/I/V) of LIR are conserved in FUNDC2 in land vertebrates and in FUN14 proteins from three invertebrates that are phylogenetically close to vertebrates, lancelet *B. floridae* (F and L), sea urchin *Strongylocentrotus purpuratus* (F and L) and sea squirt *Ciona intestinalis* (F and I), as well as the cnidarian *Nematostella vectensis* (Y and I). However, the first aromatic residue of LIR is variable in FUNDC2 in fish genomes. Two phosphorylation sites, Y18 and S13 of human FUNDC1, are completely conserved in vertebrate FUNDC1. The key site S17, phosphorylated by ULK1 is a key regulator of general autophagy (Wu et al., 2014), and is only conserved in land vertebrate FUNDC1. S17 is conserved in *Lepisosteus oculatus* and *Latimeria chalumnae*, but is substituted to L in zebrafish *D. rerio* and to V in the other four fully sequenced fish genomes (Species phylogeny in Supplementary Figure 2). The intermembrane space region from amino acids 69–73 is responsible for the direct interaction between FUNDC1 and OPA1, a mitochondrial fission/fusion protein in the intermembrane space (Chen et al., 2016). In this region, the positively charged amino acid residue K70 is important for this interaction, and the replacement of K70 with a positively charged R retained the interaction (Chen et al., 2016). K70 is conserved in nearly all FUN14 domain-containing proteins in animals and is replaced by R with very similar physicochemical properties in FUNDC1 among ray-finned fish genomes (Figure 1B). Thus, the FUNDC1-OPA1 interaction occurs in both land vertebrates and ray-finned fishes. The key site K119, which is responsible for interacting with MARCH5, is located in the cytosol-exposed region between TM2 and TM3. K119 is only conserved in FUNDC1 (Figure 1B).

The function of FUNDC2 in mammalian cells remains poorly understood, although several high-throughput experiments (Stelzl et al., 2005; Castello et al., 2012; Hein et al., 2015; Huttlin et al., 2015) determined that the interacting proteins of human FUNDC2 are involved in mitophagy (FUNDC1), apoptosis and transport between the endoplasmic reticulum and Golgi (Supplementary Table 5). We found that FUNDC2 genes in fishes probably do not have binding capability because they do not have the characteristic LIR motif (Figure 1B). In addition, FUNDC2 was lost from the amphibian lineage (Figure 3). These results are in accord with the molecular evolutionary analysis (Table 2, Supplementary Table 4), which suggests that the FUNDC1 and FUNDC2 paralogous subfamilies underwent divergent selection pressures at the sequence level during their independent evolution after WGD. Moreover, FUNDC2 genes have experienced relaxed selective pressure, which represents relaxed functional importance at some sites relative to FUNDC1. FUNDC2 is thereafter not included in the evolutionary analysis.

### Evolution of two other mitophagy receptors, BNIP3 and its homolog NIX

**Figure 5:**
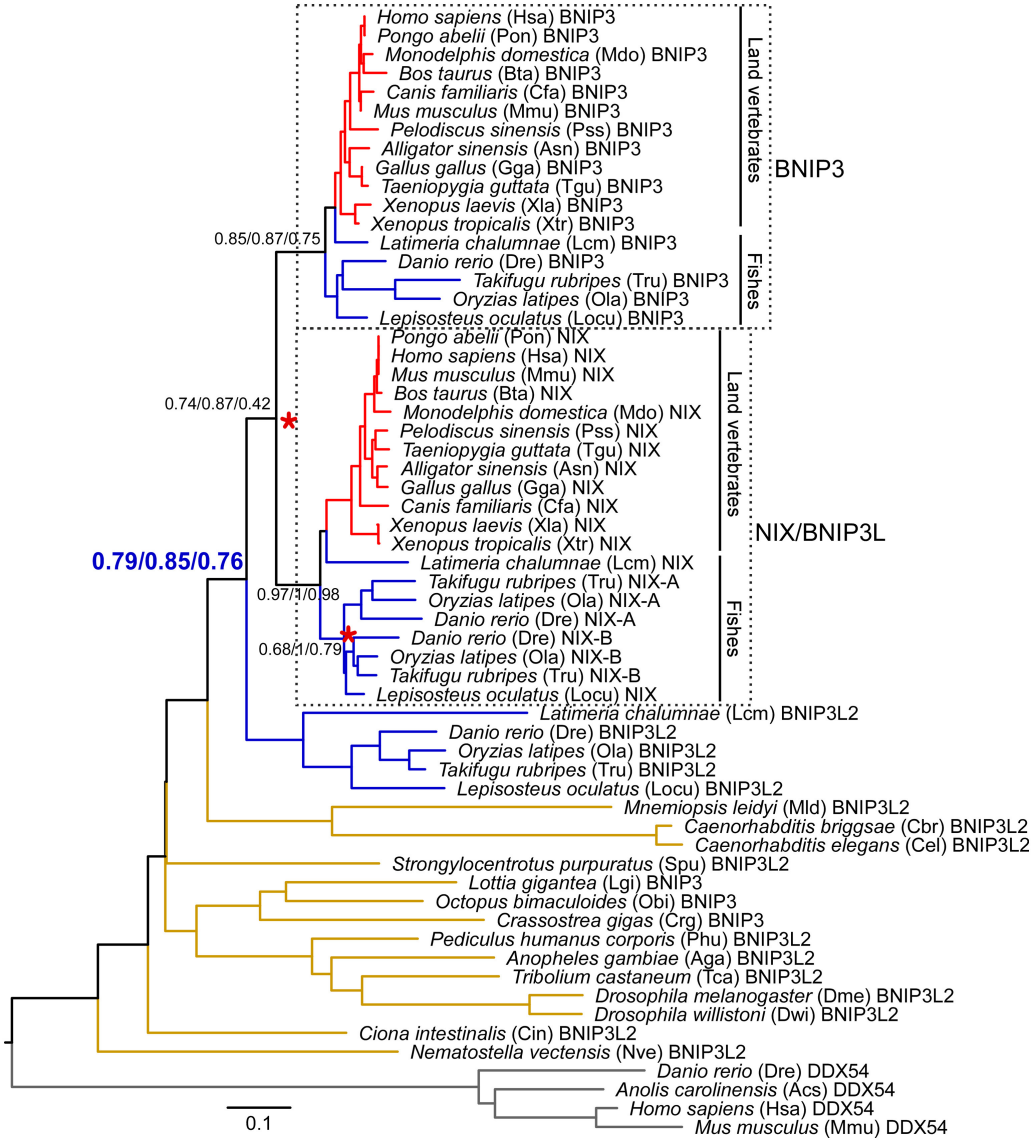
Phylogeny of BNIP3 domain-containing proteins in animals. 56 BNIP3 domain-containing proteins are present in the 32 sampled animal species. Four DDX54 proteins were set as the outgroup to root the gene tree. The full-length sequences were aligned using MAFFT with the L-INS-i method. The tree topology is derived from a neighbor-joining tree. Statistical support values corresponding to neighbor-joining bootstrap, Bayesian posterior probability, and maximum-likelihood aLRT values are shown next to the corresponding nodes. The unit of branch length is the expected fraction of amino acid substitutions. Each leaf node is depicted as a full species name, followed by a three- or four-letter abbreviation of the species name and a gene ID. Vertebrate- and teleost-specific WGDs are marked with red asterisks. Branches in red, blue, and brown denote land vertebrates, fishes, and invertebrates, respectively. Branches of BNIP3 and NIX (also named BNIP3L) in land vertebrates and fishes are also indicated with black lines. The clades clustering vertebrate BNIP3 and NIX subfamilies are enclosed within dashed boxes and are included in molecular evolutionary and functional divergence analyses. Detailed protein information is noted in Supplementary Table 1. Proteins sequences are available in Supplementary Data 1. Alignments and three trees are available in Supplementary Data 2.

BNIP3 and NIX are two other mitophagy receptors in mammals. BNIP3 induces clearance of mitochondria and the endoplasmic reticulum via autophagy under hypoxic conditions (Hanna et al., 2012), and its homolog NIX mediates mitophagy during hypoxia and during erythrocyte differentiation (Novak et al., 2010). Both proteins localize to the mitochondrial outer membrane through their C-terminal TM domains. Their N termini are exposed to the cytosol (Figure 1C). We used an HMM-based search of BNIP3 domain-containing proteins in fully sequenced eukaryotic and prokaryotic genomes (Supplementary Table 1). A total of 142 BNIP3 domain-containing proteins were found in all major metazoan subdivisions (Table 1). In contrast, no BNIP3 domain-containing sequence can be found in prokaryotes, fungi, plants or protists. The choanoflagellate *M. brevicollis*, which belongs to protists and is the closest relative of metazoans, lacks a BNIP3 domain sequence. Thus, BNIP3 domain sequences are restricted to the metazoan lineage. Phylogenetic analysis showed that in invertebrates there is only one gene copy, while there are three paralogous lineages (BNIP3, NIX and BNIP3L2) in vertebrates (Figure 5). The pattern of duplication events is likely to have resulted from two rounds of vertebrate-specific WGDs (Supplementary Figure 2). Indeed, both the Ohnologs and Synteny databases suggested that NIX and BNIP3 arose from WGD in the vertebrate ancestor (see Materials and Methods). Syntenic analysis also showed strong syntenic conservation between human chromosome 10 with BNIP3 and chromosome 8 with NIX (Supplementary Figure 3). The basal group of BNIP3 and NIX subfamilies includes another gene copy of BNIP3L2 that is only present in all ray-finned fishes and the earliest lobe-finned fish, coelacanth *L. chalumnae* (Lcm) (Figure 5, Supplementary Figure 2). In the NIX sublineage, there are two NIX copies in teleosts, which likely emerged from the teleost-specific WGD (Supplementary Figure 2), dating to approximately 350 million years ago (Meyer and Van de Peer, 2005). At the coding region level, selective pressure analysis showed that the vertebrate lineages of BNIP3 and NIX as a whole has a mean ω value of 0.081 (one-ratio model), reflecting strong purifying selection to act against changes during evolution. BNIP3 and NIX subfamilies underwent asymmetrical evolution (*P* = 2.05E-03 from two-ratio branch models in Supplementary Table 6), which is similar to FUNDC1 and FUNDC2 in vertebrates. However, BNIP3 and NIX subfamilies did not show divergent selection pressure (*P* = 0.21 in Supplementary Table 7) after gene duplication, which is in conformity with their similar functions in apoptosis (Zhang and Ney, 2009; Ney, 2015) and mitophagy (Wei et al., 2015).

The alignment of BNIP3 domain-containing sequences from invertebrates to human revealed that the four residues of the BNIP3 LIR motif (WVEL) are conserved in all homologous sequences (Figure 1D). Two phosphorylation sites at serine residues 17 and 24, which are phosphorylated by an unknown kinase and flank the BNIP3 LIR, regulate the binding of BNIP3 to LC3B and GATE-16 (Wu et al., 2014). S17 of human BNIP3 is conserved in all BNIP3 domain-containing proteins in animals, whereas S24 is only conserved in BNIP3 among land vertebrates and several fish genomes (Figure 1D). A novel short linear motif (SLiM as MEKILLDAQHE in human NIX) in the cytoplasmic region of NIX (Figure 1C) is essential for mitochondrial clearance in reticulocytes (Zhang et al., 2012). The SLiM is an 11-amino acid motif containing a triplet of hydrophobic residues at its center and charged residues (Lys/Arg/Asp/Glu) flanking the triplet. The SLiM is conserved among the BNIP3 and NIX sublineages as well as the teleost fish branch of BNIP3L2 (Figure 1D). Mutation of the central leucine (Leu) residue at position 75 in the triplet of human NIX abolishes all activity of NIX (Zhang et al., 2012). L75 of human NIX is conserved in the BNIP3 domain-containing proteins of all species except nematodes and ctenophores (Figure 1D).

### Evolution of proteins in FUNDC1 pathway

**Figure 6:**
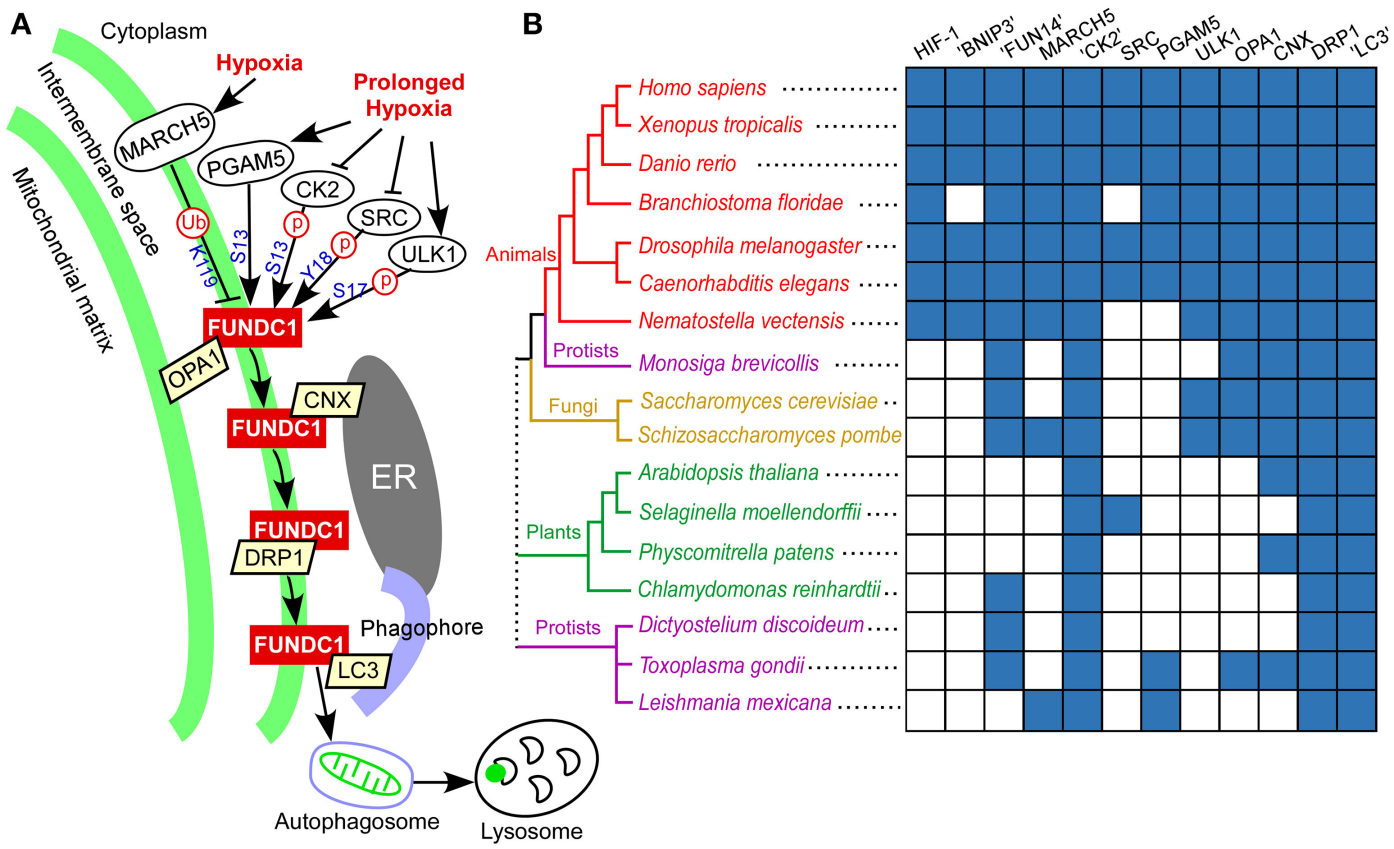
FUNDC1 pathway in response to hypoxia and phylogenetic profiles of genes in this pathway. **(A)** Model of FUNDC1 pathway for mitochondrial fission and mitophagy in response to hypoxia in mammalian cells. Under normal (unstressed) conditions, FUNDC1 interacts with OPA1, a mitochondrial fusion protein in the intermembrane space (Chen et al., 2016), and is inactivated by phosphorylation at S13 by CK2 and at Y18 by SRC kinase (Liu et al., 2012; Chen et al., 2014). The initial hypoxic stress increases the interaction between MARCH5 and FUNDC1, leading to FUNDC1 ubiquitylation at K119 and degradation. Degradation of FUNDC1 by MARCH5 desensitizes mitochondria to hypoxia-induced mitophagy. Under prolonged hypoxic conditions, OPA1, CK2, and SRC kinase dissociate from FUNDC1. Dephosphorylation at Y18 plays a critical role in reactivating the FUNDC1-mediated mitophagy (Kuang et al., 2016). The PGAM5-mediated dephosphorylation at S13 (Chen et al., 2014) and ULK1-mediated phosphorylation at S17 (Wu et al., 2014) add additional regulations. At the early stage of hypoxia, FUNDC1 increasingly accumulates in the mitochondrion associated membrane and interacts with the endoplasmic reticulum protein calnexin (CNX). As mitophagy proceeds, FUNDC1 dissociates from calnexin, binds to DRP1 in the mitochondrion associated membrane and triggers mitochondrial fission (Wu et al., 2016). Finally, FUNDC1 binds to LC3 on the phagophore, thereby aiding engulfment of the defective mitochondrion in an autophagosome. The autophagosome then fuses with lysosomes to allow degradation and recycling of mitochondria to occur. **(B)** Distribution of human genes in the FUNDC1 pathway as well as two other mitophagy receptors (NIX and BNIP3) and their transcription factor (HIF-1α) (listed on the top) under hypoxia in a representative group of fully sequenced eukaryotic genomes (on the left). “CK2” includes CK2 catalytic subunits (α1 and α2). “LC3” includes LC3 and related members of the GABARAP family. “FUN14” and “BNIP3” were respectively identified using a HMM-based search of the FUN14 domain and BNIP3 domain. DRP1 and OPA1 were identified and classified according to the phylogenetic analysis of dynamin domain-containing proteins (i.e., dynamin superfamily) (Supplementary Figure 4). Other gene families were identified using a modified bidirectional BLASTP method (see Materials and Methods). The presence of a homolog of a human gene family in a particular species is in blue, and the lack of an apparent homolog is in white. A consensus cladogram showing the topology (branch lengths are not intended to be to scale) of the phylogenetic relationships between the species is displayed. Colored branches and nodes indicate membership in different major lineages of the tree. The dotted line joining the major branches indicates an unclear phylogenetic relationship.

Being regulated by post-translational modifications, FUNDC1 interacts with other proteins to mediate mitochondrial fission and mitophagy in response to hypoxia stress (Figure 6A). To understand how the FUNDC1 pathway evolved, the components of this signaling pathway were mapped along the eukaryotic evolutionary line (Figure 6B). DRP1 and OPA1 were classified according to the phylogeny of dynamin domain-containing proteins (Supplementary Figure 4). CK2, DRP1 and “LC3” (LC3 and related members of the GABARAP family) are present in nearly all complete eukaryotic genomes. Except for CK2, the regulators of FUNDC1 are distributed mainly in animals. In addition to animals, the regulators of FUNDC1 are also present in some fungi (MARCH5 and ULK1), some protozoan genomes (MARCH5 and PGAM5) and a few lower plants (PGAM5 and SRC in several green algae and the earliest vascular plant, *Selaginella moellendorffii*). Calnexin (CNX) is in all major eukaryotic branches except for some protists. OPA1, the mitochondrial fission/fusion protein, is in animals, fungi and some protists, including a choanoflagellate *M. brevicollis*. Thus, “FUN14” co-appears with its downstream binding components (OPA1, calnexin, DRP1 and “LC3”) in fungi, *M. brevicollis* and animals. Both BNIP3 and NIX are transcriptionally regulated by HIF-1α in response to hypoxia stress. BNIP3, NIX and HIF-1α are only present in animals (Figure 6B), and are absent from *M. brevicollis* (see Materials and Methods), indicating their origins within the early metazoan evolution.

### Variable substitution patterns of the mitophagy receptors and their interacting proteins in fishes and mammals

Is there any variable pattern in the molecular evolution of FUNDC1, BNIP3 and NIX in teleost fishes, a major water-breathing vertebrate lineage that makes up 96% of all fish, and in mammals, a major air-breathing vertebrate lineage? For BNIP3 and NIX, the overall dN values (the numbers of synonymous substitutions per synonymous site) along the sequences were three times higher (0.137 vs. 0.044 for BNIP3 and 0.152 vs. 0.054 for NIX in Table 3) in teleost fishes than in mammals. ω ratio measures the selection pressure acting on a protein sequence. The average ω ratios of teleost fishes were 2.4 (0.095 vs. 0.039 in Table 3) and 1.7 (0.085 vs. 0.049 in Table 3) times higher than those of mammals for BNIP3 and NIX, respectively, and the difference was significant through LRTs in the CODEML program (Supplementary Table 8) (see Materials and Methods). Interestingly, BNIP3 and NIX have a similar substitution pattern with their transcription factor HIF-1α (Rytkonen et al., 2008) (Supplementary Table 9). FUNDC1 showed a different substitution pattern. The overall ω ratio along full-length FUNDC1 sequences in mammals (0.052 in Table 3) was marginally (1.3 times) higher than that in teleost fishes (0.04). We then divided the FUNDC1 sequence into three domain partitions, and investigated the substitution pattern for each domain. The ω ratios for individual FUNDC1 domain partitions were between 1.7 and 2.6 (calculated from Table 4) times higher in mammals than those in teleost fishes.

**Table 3:** Estimation of dN and dS substitution rate variation across the entire gene sequences of FUNDC1, MARCH5, BNIP3, and NIX in the lineages of teleost fishes, mammals and land vertebrates.

**Gene**	**Lineage^a^**	**dN^b^**	**dS^b^**	**ω (dN/dS)^b^**
FUNDC1	Teleost fishes	0.055	1.191	0.040
	Mammals	0.027	0.456	**0.052**
	Land vertebrates	0.066	1.011	**0.060**
MARCH5	Teleost fishes	1.224	0.019	0.016
	Mammals	0.280	0.022	**0.024**
	Land vertebrates	0.694	0.019	**0.020**
BNIP3	Teleost fishes	0.137	1.341	**0.095**
	Mammals	0.044	1.100	0.039
	Land vertebrates	0.080	1.598	0.041
NIX	Teleost fishes	0.152	1.602	**0.085**
	Mammals	0.054	0.710	0.049
	Land vertebrates	0.075	1.267	0.041

a *The relationship between teleost fishes and land vertebrates is displayed in Supplementary Figure 2*.

b *dN means the number of synonymous substitutions per synonymous site and dS means the number of non-synonymous substitutions per non-synonymous site. dN and dS were estimated from the SNAP program (see Materials and Methods). ω estimates the relative rate of non-synonymous and synonymous substitutions (dN/dS). The ω value in teleost fishes was compared with those in mammals and land vertebrates, and the higher ω value is shown in bold*.

**Table 4:** Estimation of substitution rate variation on domains of FUNDC1 and MARCH5 in the lineages of teleost fishes, mammals and land vertebrates.

**Gene**	**Domain^a^**	**Teleost fishes^b^**	**Mammals^b^**	**Land vertebrates^b^**
FUNDC1	Cyto1 (1–49 aa)	0.020	**0.051**	**0.067**
	Mito (50–96 aa)	0.023	**0.038**	**0.027**
	Cyto2 (97–155 aa)	0.023	**0.050**	**0.056**
MARCH5	Cyto1 (1–96 aa)	0.041	0.066	0.040
	Mito1 (97–138 aa)	**0.027**	0.043	0.022
	Cyto2 (139–228 aa)	0.005	**0.020**	**0.008**
	Cyto3 (238–278 aa)	0.015	**0.070**	**0.018**

a *Domain regions of FUNDC1 are displayed in Figure 1A and those of MARCH5 are shown in Supplementary Figure 6A*.

b *The ω value (=dN/dS) in teleost fishes was compared with those in mammals and land vertebrates, and the higher ω value is in bold. dN and dS were estimated from the SNAP program*.

We were also interested to know which of the proteins in the FUNDC1 pathway presented a similar substitution pattern to FUDNC1. MARCH5 (Supplementary Figure 5), a novel feedback regulator of FUNDC1 that specifically ubiquitylates and degrades FUNDC1 in response to hypoxic stress (Chen et al., 2017), showed the same pattern as FUNDC1. The ω ratios were between 1.5 and 4.7 times higher in mammals than in teleost fishes at the levels of the full-length MARCH5 sequence (Table 3, Supplementary Table 8) and the three cytosol-exposed domain regions (Table 4, Supplementary Figure 6A). FUNDC1 interacts with the MARCH5 Cyto2 through K119 (Chen et al., 2017). K119 is in FUNDC1 Cyto2 as shown in Table 4, Figure 1A. OPA1 has higher rates of substitution in mammals than in teleost fishes in only the coiled-coil domain (Supplementary Tables 9, 10, Supplementary Figure 6B). Other components in the FUNDC1 pathway presented higher ω ratios in teleost fishes (Supplementary Table 9), which is similar to BNIP3 and NIX. In land vertebrates the average ω ratios of the three mitophagy receptors and their interacting proteins were approximately the same as those in the mammalian dataset (Tables 3, 4, Supplementary Tables 8–10).

## Discussion

### When did the modern FUNDC1 and BNIP3 originate in response to hypoxia?

Our study revealed that FUN14 domain-containing proteins are present in archaeal, bacterial and eukaryotic genomes (Figure 2, Supplementary Figure 1). FUN14 proteins were found in six bacterial phyla, but not in Alphaproteobacteria. Mitochondria descended from endosymbiosis of a single alphaproteobacterial ancestor, the so-called protomitochondrion which is an ATP-consuming “compartment” (Gray et al., 1999). The family of BNIP3 domain-containing proteins was established in early metazoan evolution (Figures 5, 6B). In yeast, Atg32 has been identified as the mitophagy receptor that is required for selectively targeting mitochondria for autophagy in response to oxidative stress, and this protein is not required for non-selective autophagy (Kanki et al., 2009; Okamoto et al., 2009). Interestingly, no mammalian homolog of Atg32 has been identified. The function and regulation of FUNDC1 strongly resemble those of Atg32 (Wei et al., 2015). Out study found that both FUN14 domain- and BNIP3 domain-containing proteins are absent from land plants (Figure 6B). Thus, it seems that there are different sets of mitophagy receptors under hypoxia conditions in yeast, mammals and land plants. Although the molecular mechanism for mitophagy under hypoxia in land plants is still poorly understood (Minibayeva et al., 2012), we hypothesize that plant cells probably use different receptors or different mechanisms for clearing mitochondria to survive under hypoxic stress.

During metazoan evolution, when did the three mitophagy receptors, FUNDC1, BNIP3 and NIX, acquire the ability to bind to LC3 for mediating mitochondrial removal? The amino acid residues D19, K49 and L53 of human LC3B, which respectively contact key residues Y18, S17 and V20 (Wu et al., 2014; Kuang et al., 2016; Lv et al., 2017) of human FUNDC1, are nearly (D19) or completely (K49 and L53) conserved in all animals (Supplementary Figure 7). The regulators for ubiquitylating, phosphorylating and dephosphorylating human FUNDC1 are conserved in nearly all eukaryotes (CK2), or they appear mainly in animals (MARCH5, ULK1, PGAM5, and SRC) (Figure 6). Furthermore, we tried to answer this question by placing the functionally important residues of human FUNDC1 and BNIP3 across metazoan species (Figures 1B,D). All of the key residues (marked as red asterisks in Figure 1B) of human FUNDC1 are completely conserved in land vertebrate FUNDC1, which has also been noted by the Chen lab (Wei et al., 2015). This result suggests that the function of FUNDC1 as a mitophagy receptor under hypoxia is not limited to mammals, but it can be expanded across land vertebrates. Key residues (Y18 and L21) in the typical LIR pattern of Y/W/F-x-x-L/I/V and two of the key phosphorylation sites (Y18 and S13) are conserved in FUNDC1 in both land vertebrates and fishes, but they are variable in invertebrates. The unphosphorylated state of Y18 in the LIR motif serves as a unique molecular switch for mitophagy in mammalian cells (Kuang et al., 2016). Thus, we consider that FUNDC1 in fish genomes is still capable of binding to LC3 via LIR in response to hypoxia. However, the corresponding amino acid S17 in human FUNDC1 is Leu or Val in fish FUNDC1, which cannot be phosphorylated by ULK1, indicating that fish FUNDC1 lacks the general layer of regulation by ULK1.

For BNIP3 and NIX, because LIR motifs and one phosphorylation site (S17 of human BNIP3) are completely conserved in all BNIP3 domain-containing proteins including the nematode *Caenorhabditis elegans* (Figure 1D), the ability of BNIP3 and NIX to bind to LC3 may have evolved in the early evolution of animals. Indeed, DCT-1, the *C. elegans* homolog of mammalian BNIP3 and NIX, was reported to be a key mediator of mitophagy promoting longevity under stress, but the role of DCT-1 in hypoxia-induced mitophagy and whether HIF-1 regulates DCT-1 remain to be determined (Palikaras et al., 2015). However, the other phosphorylation site (S24 of human BNIP3) is only completely conserved in BNIP3 among land vertebrates and is variable in BNIP3 from fishes. It suggests that while BNIP3 in vertebrates bind to LC3 through the regulation by phosphorylating S17 and S24, some fish genomes lack the layer of regulation at S24.

In conclusion, we postulate that FUNDC1 may have already gained the ability to bind to LC3 controlled by post-translational regulations during the early evolution of vertebrates, while the evolutionary origin of BINP3 and NIX binding to LC3 dates far back to the early invertebrates, such as nematodes.

### Atmospheric oxygen levels as a selection force in the development of hypoxia-induced mitophagy

FUNDC1 and BNIP3 genes in fishes lack a layer of regulation by phosphorylating S17 and S24, respectively, which are conserved in land vertebrates. It is interesting to explain the evolutionary pressure driving the slight difference in molecular mechanism at the level of post-translational regulations between fishes and land vertebrates. HIF-1α is a primary oxygen sensing protein and targets a number of proteins including BNIP3 and NIX. The substitution rate of HIF-1α in teleost fishes was two times higher than that in mammals (Rytkonen et al., 2008). Fishes experience a tenser oxygen level than land vertebrates because water contains only 1/30th of the oxygen compared to the same volume of air at the same partial pressure. Thus, the different oxygen tensions in water that are experienced by fish may have had a differential impact on the evolution of HIF-1α (Taylor and McElwain, 2010). BNIP3 and NIX showed the same substitution pattern as HIF-1α (Table 3, Supplementary Tables 8, 9), indicating that the evolution of BNIP3 and NIX, similar to their transcriptional factor HIF-1α, might also be under a selection force driven by the different oxygen concentrations in water than those in air.

Interestingly, compared with BNIP3/NIX/HIF-1α, FUNDC1 and its feedback regulator MARCH5 present a reverse substitution pattern at both the full-length level (Table 3, Supplementary Table 8) and the domain level (Table 4), especially in cytosol-exposed domains. The MARCH5/FUNDC1 axis desensitizes mitochondrial degradation under the initial hypoxic stress and avoids improper clearance of undamaged mitochondria through mitophagy (Figure 6A). MARCH5 uniquely regulates the level of FUNDC1 in fine-tuning hypoxia-induced mitophagy, as MARCH5 failed to regulate the level of NIX (Chen et al., 2017). Atmospheric oxygen concentrations varied throughout metazoan evolution (Falkowski et al., 2005; Taylor and McElwain, 2010). Animals evolved to utilize the chemical reduction of molecular oxygen as the main source of metabolic energy of the cell, making an adequate oxygen supply a key to survival (Taylor and McElwain, 2010). The fluctuating levels and the overall rise of atmospheric oxygen have been linked to key stages in land vertebrate evolution, including the invasion of land by vertebrates, and the radiation and subsequent increase in the average size of various mammals (Falkowski et al., 2005). These dynamic developmental processes in land vertebrate evolution may support our hypothesis that fluctuating oxygen concentrations might have an important role in selectively driving the evolution of FUNDC1, which specifically regulates mitophagy under oxygen deprivation, and MARCH5, which fine-tunes hypoxia-induced mitophagy.

Some invertebrates, such as molluscs and nematodes, can adapt to environments under a very low pressure of oxygen. Most molluscs are aquatic, but some are terrestrial. The origin of molluscs probably dates back to the late Precambrian (Fedonkin and Waggoner, 1997). Thereafter, the mollusc crown group radiated rapidly during the Cambrian explosion approximately 540 million years ago (Vinther, 2015), before the strong increase of oxygen in earth. Our results showed that both the LIR motif of FUNDC1, which is essential for FUNDC1 binding to LC3, and the SLiM motif of NIX, which is essential for NIX's role of programmed mitochondrial clearance during erythrocyte differentiation, are not conserved in most invertebrates, including molluscs and nematodes (Figure 1). This observation suggests that invertebrates that adapt to environments with low oxygen tensions might utilize alternative ways to carry out the specific roles of FUNDC1 and NIX to maintain proper mitochondrial homeostasis. For example, nematodes can develop and survive in low oxygen levels through anaerobic respiration and the maintenance of mitochondrial signatures, e.g., rhodoquinone (Gems, 2009).

FUNDC1 induces mitophagy under hypoxia using a different mechanism from that of BNIP3 and NIX (Ding and Yin, 2012; Liu et al., 2012; Ney, 2015). mRNA expression levels of NIX and BNIP3 are increased by HIF-1α under hypoxia, but the mRNA level of FUNDC1 is decreased. In human BNIP3, serine phosphorylation increases BNIP3-LC3 binding affinity, but at FUNDC1, it is unusual for dephosphorylation events at Y18 and S13 to induce its binding to LC3. Moreover, NIX and BNIP3 localize to other organelles and are involved in regulating apoptosis by affecting mitochondrial respiration, whereas FUNDC1 plays a specific role in hypoxia-induced mitophagy. In this study, by placing these receptors in a molecular evolutionary context, we found that the FUN14 domain- and BNIP3 domain-containing protein families also show different selection pressures exerted on the duplicated subfamilies after WGDs and different substitution patterns in teleost fishes and mammals. Little is known about the transcriptional regulation of FUNDC1 during hypoxia, and it is still unclear whether FUNDC1 is a target of HIF-1α (Wei et al., 2015). Thus, the hypoxia-induced mitophagy regulated by FUDNC1/MARCH5 seems distinct from the HIF-1α-dependent mitophagy regulated by BNIP3/NIX in terms of mRNA expression patterns, responses to hypoxia, and patterns in the molecular evolution of water- and air-breathing vertebrate lineages.

## Author contributions

XW contributed to the conception and design of the research, conducted phylogenetic and molecular evolutionary analysis, and wrote the manuscript. FW conducted key residue analysis, performed syntenic cluster analysis and interpreted the results. QW, SC, SZ, and MS participated in generating the figures and interpreting the results. All authors revised the manuscript and approved the final version of the manuscript.

### Conflict of interest statement

The authors declare that the research was conducted in the absence of any commercial or financial relationships that could be construed as a potential conflict of interest.

## Abbreviations

ROS: reactive oxygen species
BNIP3: Bcl-2/adenovirus E18 19-kDa-interacting protein 3
HIF-1α: hypoxia-inducible factor 1 α
BH3: Bcl-2 homology 3
LC3: microtubule-associated protein 1 light chain 3
LIR: LC3 interaction region
FUNDC1: FUN14 domain-containing protein 1
WGD: whole-genome duplication
HMM: Hidden Markov Model
LRT: likelihood ratio test
TM: transmembrane domain
CNX: Calnexin
dS: the number of synonymous substitutions per synonymous site
dN: the number of non-synonymous substitutions per non-synonymous site
ω ratio: dN/dS.
