# Supplementary material for: Phylogenetic and Molecular Evolutionary Analysis of Mitophagy Receptors under Hypoxic Conditions
Source: Front Physiol. 2017 Jul 26;8:539. doi: 10.3389/fphys.2017.00539 (PMC5526904; doi:10.3389/fphys.2017.00539)
Supplement: Supplementary file 2 [file SupplementaryMaterials.DOC]

***Supplementary Materials***

# Phylogenetic and Molecular Evolutionary Analysis of Mitophagy Receptors under Hypoxic Conditions

**Xiaomei Wu*****, Fei-Hua Wu, Qianrong Wu, Shu Zhang, Suping Chen, Matthew Sima**

*** Correspondence:** Xiaomei Wu, wuxm07@gmail.com

**Table of Content in this File**

Supplementary Figures 1-7

Supplementary Tables 2-10

Supplementary Note 1

Supplementary References

**Other supplementary data in separate files.**

Supplementary Table 1: The excel file entitled “Supplementary Table 1 all homolog list” contains information on (a) 217 FUN14 domain-containing proteins from 145 eukaryotic genomes (see Figure 2 and Supplementary Figure 1), (b) 80 FUN14 domain-containing proteins from 79 archaeal and bacterial genomes (see Figure 2 and Supplementary Figure 1), (c) 142 BNIP3 domain-containing proteins from 81 metazoan genomes, and (d) 185 MARCH5 homologs from 128 metazoan, fungal and protozoan genomes.

Supplementary Data 1: Amino acid sequences of all the proteins in Supplementary Table 1 in FASTA format.

Supplementary Data 2: Multiple sequence alignments and Bayesian, maximum-likelihood and neighbor-joining trees of proteins from sampled species, representing a wide variety of metazoan lineages, for phylogenetic analysis. The subset includes (a) 53 FUN14 domain-containing proteins from 33 metazoan genomes and 3 protozoan genomes (see Figure 3), (b) 56 BNIP3 domain-containing proteins from 32 metazoan genomes and four DDX54 proteins (see Figure 5), and (c) 44 MARCH5 homologs from 32 animals, two fungi and two protists (see Supplementary Figure 4).


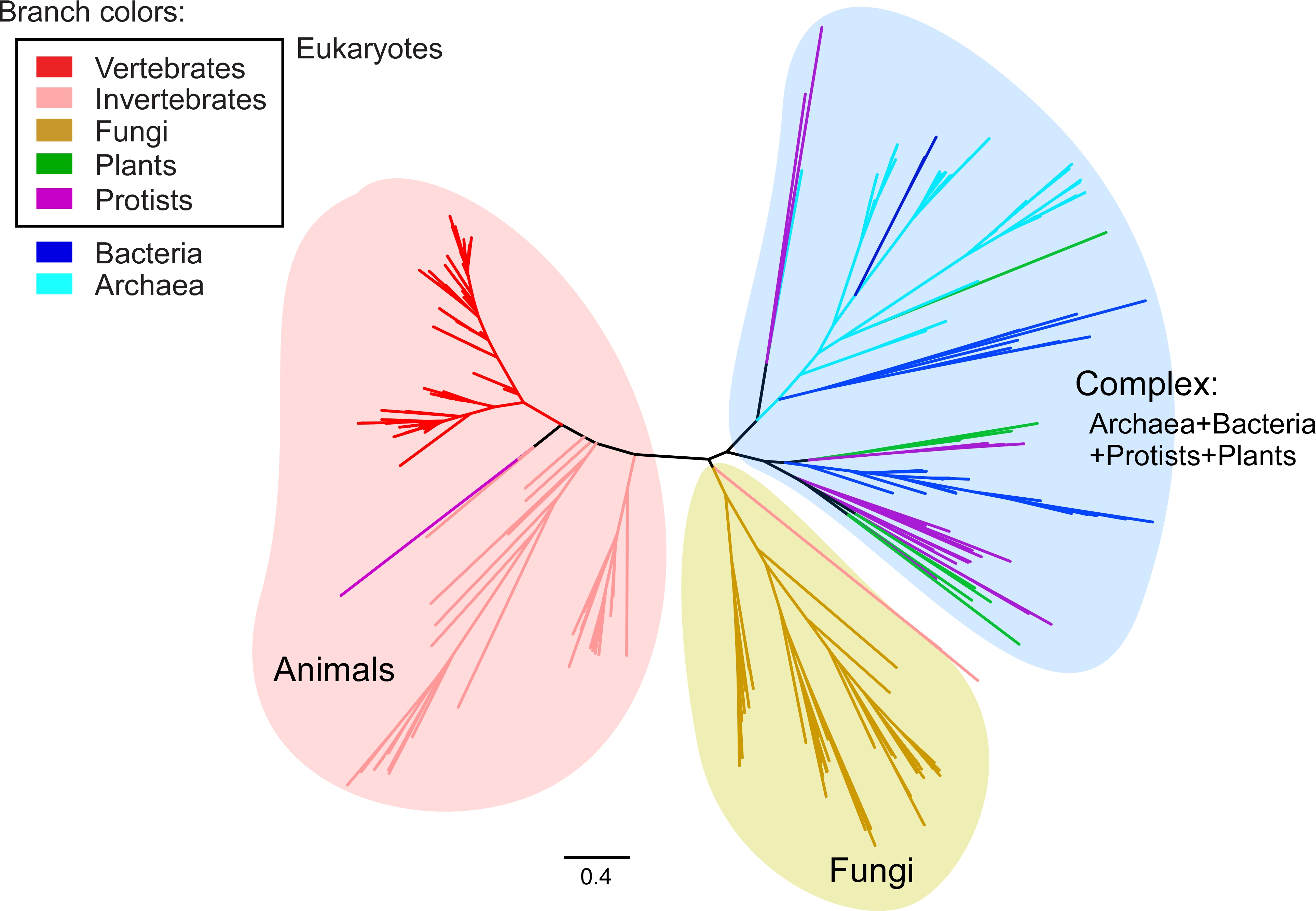


**Supplementary Figure 1. Phylogeny of 297 full-length FUN14 domain-containing proteins in all three domains of life using FastTree.** FastTree implements an ultrafast and fairly accurate approximate maximum-likelihood method . The tree was based on the same sequence alignments as in Figure 1. Branch lengths indicate the number of amino acid substitutions per site. Colored branches indicate different species lineages. Three major clades were identified, which are a metazoan clade, a fungal clade and a complex clade that includes FUN14 proteins from plants, protists, bacteria and archaea. Detailed information on these 297 FUN14 proteins is listed in Supplementary Table 1 and their amino acid sequences in FASTA format are available in Supplementary Data 1.


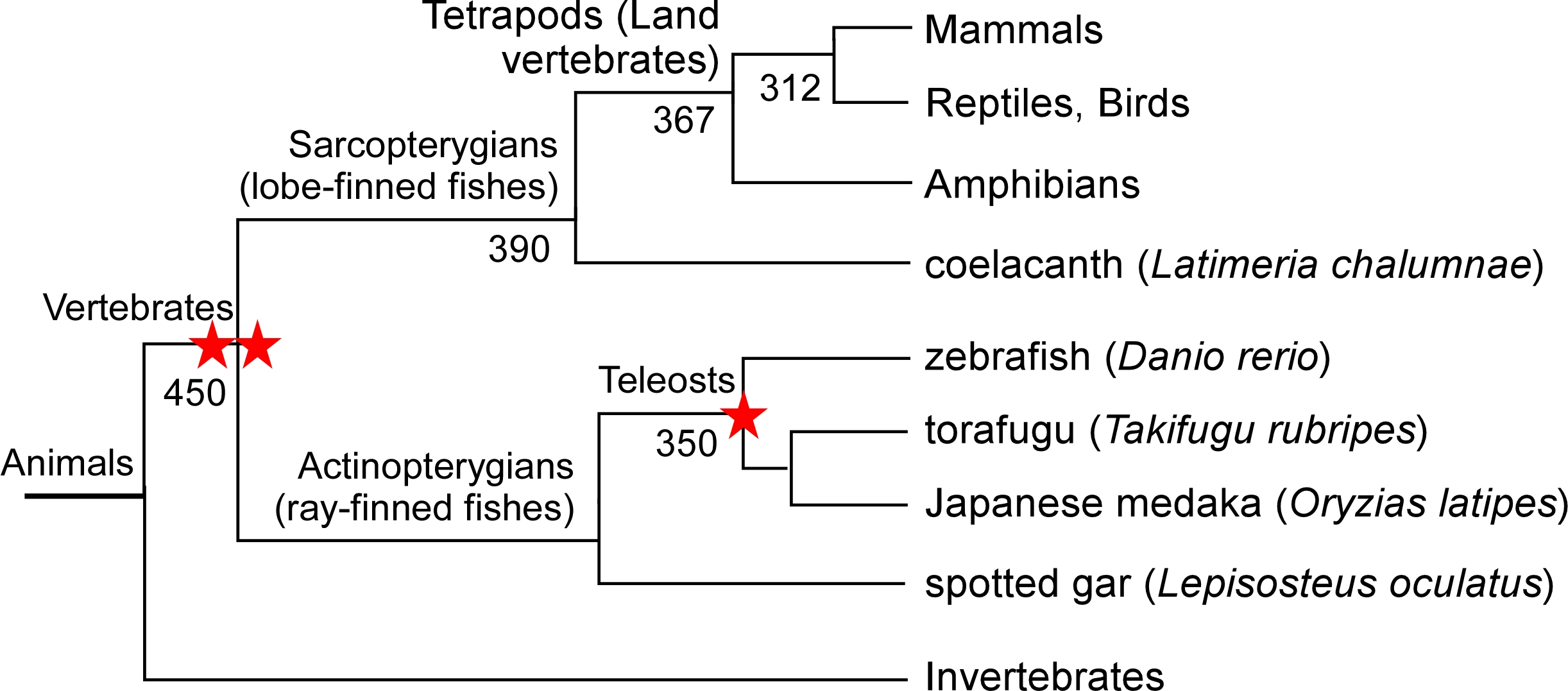


**Supplementary Figure 2. Phylogeny of the vertebrate lineage.** The phylogenetic relationship among spotted gar, coelacanth, teleosts and land vertebrates was derived from . Approximate timings of divergence (million years ago) are displayed beside the nodes . Two rounds of whole-genome duplications leading to all vertebrates and the third genome duplication leading to teleosts are indicated with red asterisks.


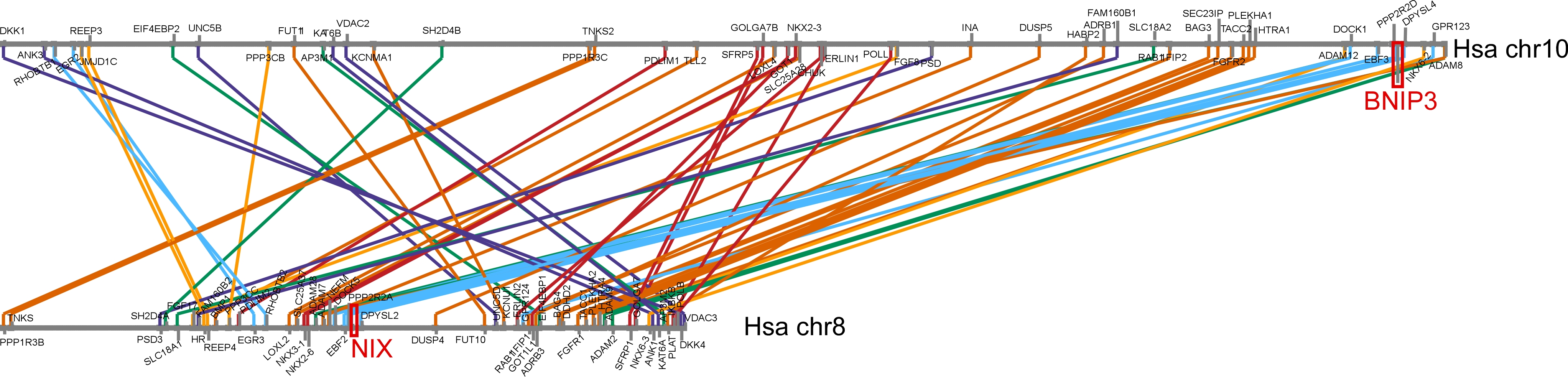


**Supplementary Figure 3. Syntenic analysis among BNIP3 and NIX in human.** The plot was obtained from the Synteny Database (<http://syntenydb.uoregon.edu/synteny_db/>). The BNIP3 and NIX paralogous syntenic clusters show strong syntenic conservation between human chromosome 10 (chr10) with BNIP3 and human chromosome 8 (chr8) with NIX. *Ciona intestinalis* (sea squirt) was used as an unduplicated outgroup and a sliding window size of 100 genes was selected. Different syntenic blocks were drawn in different colors. 59 gene pairs are shared in the clusters.


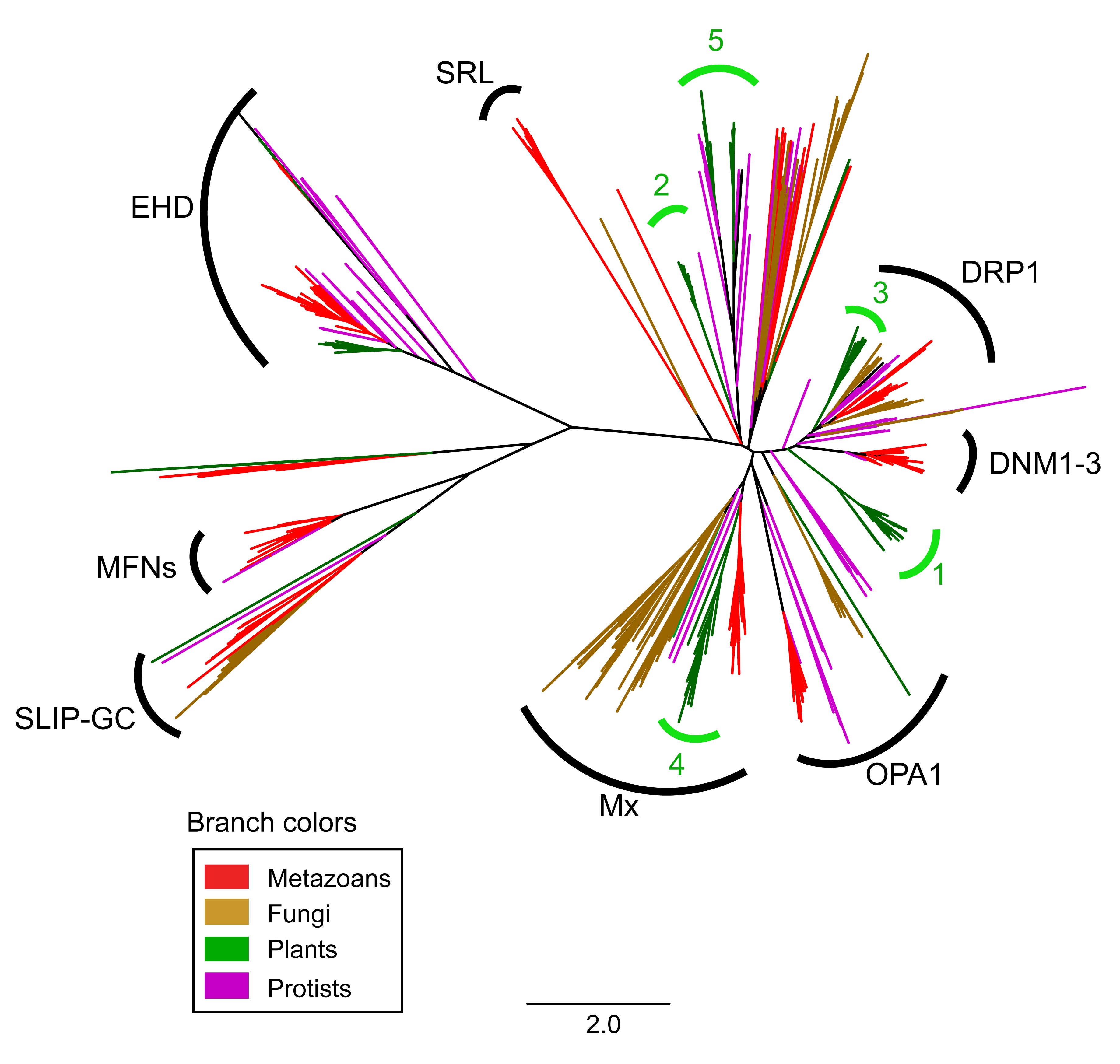


**Supplementary Figure 4. Phylogeny of dynamin domain-containing proteins in all fully sequenced eukaryotic genomes.** An HMM-based search for the dynamin domain (PF00350) was carried out in fully sequenced eukaryotic genomes. Sequences with length between 300 and 1500 aa, and covering equal to or more than 50% of the query domain were kept. Finally, 2352 total dynamin domain-containing protein sequences were obtained and aligned using MUSCLE v3.8 with the maximum number of iterations set as 2. The phylogeny was reconstructed using the FastTree program. Branch lengths indicate the number of amino acid substitutions per site. Colored branches indicate different eukaryotic species lineages. Eleven major clades are identified by their names: DNM1-3 (dynamins 1-3), DRP1 (dynamin-1-like protein), OPA1 (mitochondrial dynamin like GTPase), Mx (interferon-induced GTP-binding protein Mx1 and Mx2), SLIP-GC, MFNs (mitofusins 1 and 2), EHD (EH domain-containing proteins 1-4) and SRL (sarcalumenin precursor) highlighted in black, as well as plant clades 1 (phragmoplastin-like DRPs), 2 (plant dynamins) and 5 (chloroplast-associated DRPs) named according to Arabidopsis dynamin-related large GTPases and highlighted in green. Two other plant clades 3 (mitochondria-associated DRPs) and 4 (Mx-like DRPs) belong to the DRP and Mx clades, respectively. The branches not included in the aforementioned major clades are unclassified.


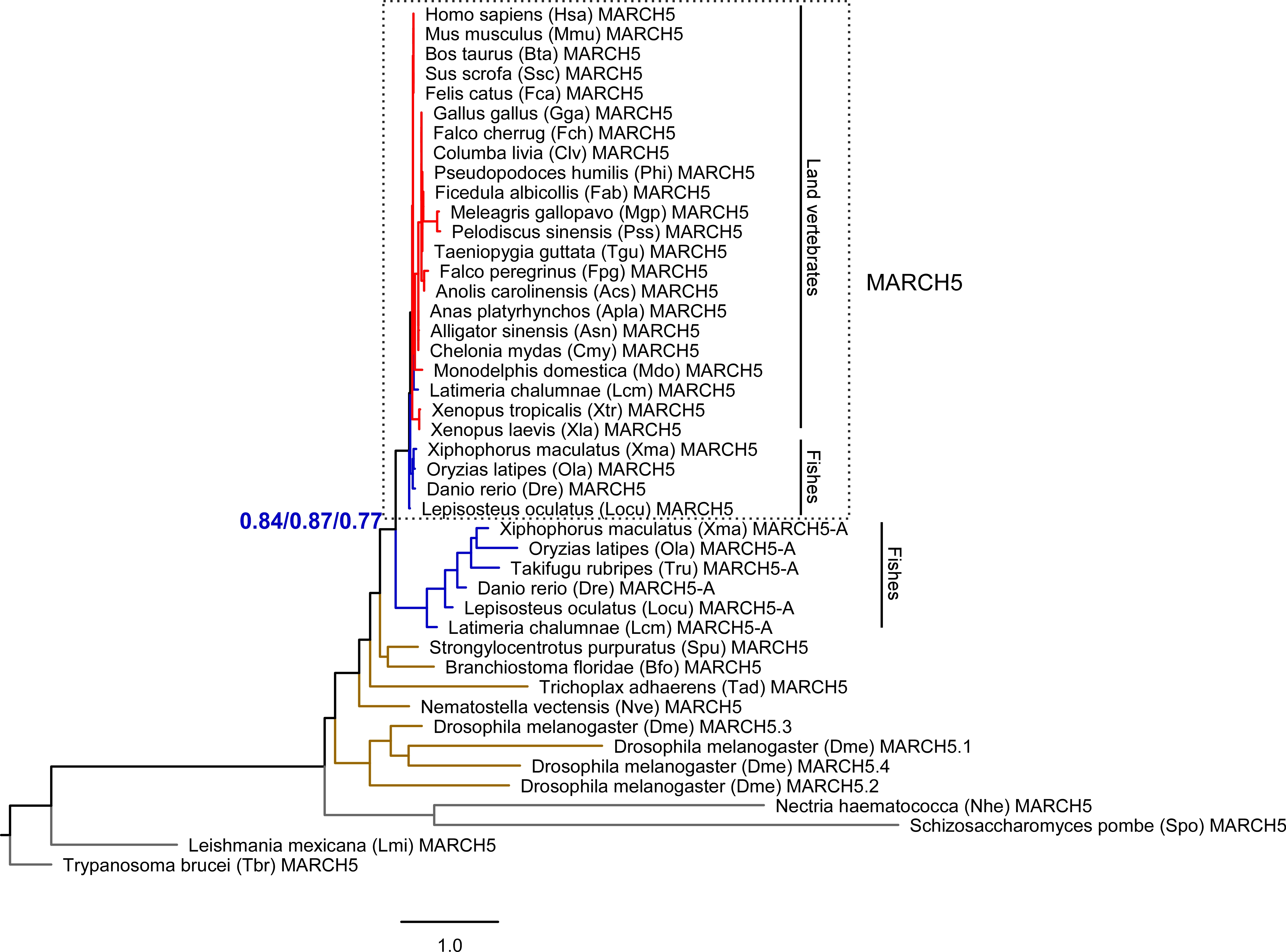


**Supplementary Figure 5. Phylogeny of MARCH5 homologs in animals.** The tree includes 44 MARCh5 proteins from 32 sampled animal species representing a wide variety of metazoan lineages, two fungal genomes and two protozoan genomes. Fungal (*Nectria haematococca* and *Schizosaccharomyces pombe*) and Protozoan (*Leishmania mexicana* and *Trypanosoma brucei*) homologs were used as outgroup to root the gene tree. The sequences were aligned using MAFFT with the L-INS-i method. The tree topology is derived from a maximum-likelihood tree. The unit of branch length is the expected fraction of amino acid substitutions per site. Each leaf node is depicted as a full species name, followed by a three- or four-letter abbreviation of the species name and a gene ID. Branches in red, blue and brown denote land vertebrates, fishes and invertebrates, respectively. Branches in land vertebrates and fishes are also indicated with black lines. Statistical support values corresponding to neighbor-joining bootstrap (0.84), Bayesian posterior probability (0.87) and maximum-likelihood aLRT (0.77) confidently unveil a split between the vertebrate clade of MARCH5 genes and a fish clade of ‘MARCH5-A’ genes at the ancestor of vertebrate. The clade of MARCH5 across vertebrates is enclosed within a dashed box and input to molecular evolutionary analysis. Detailed protein information is noted in Supplementary Table 1. Proteins sequences are available in Supplementary Data 1. Alignments and three trees are available in Supplementary Data 2.


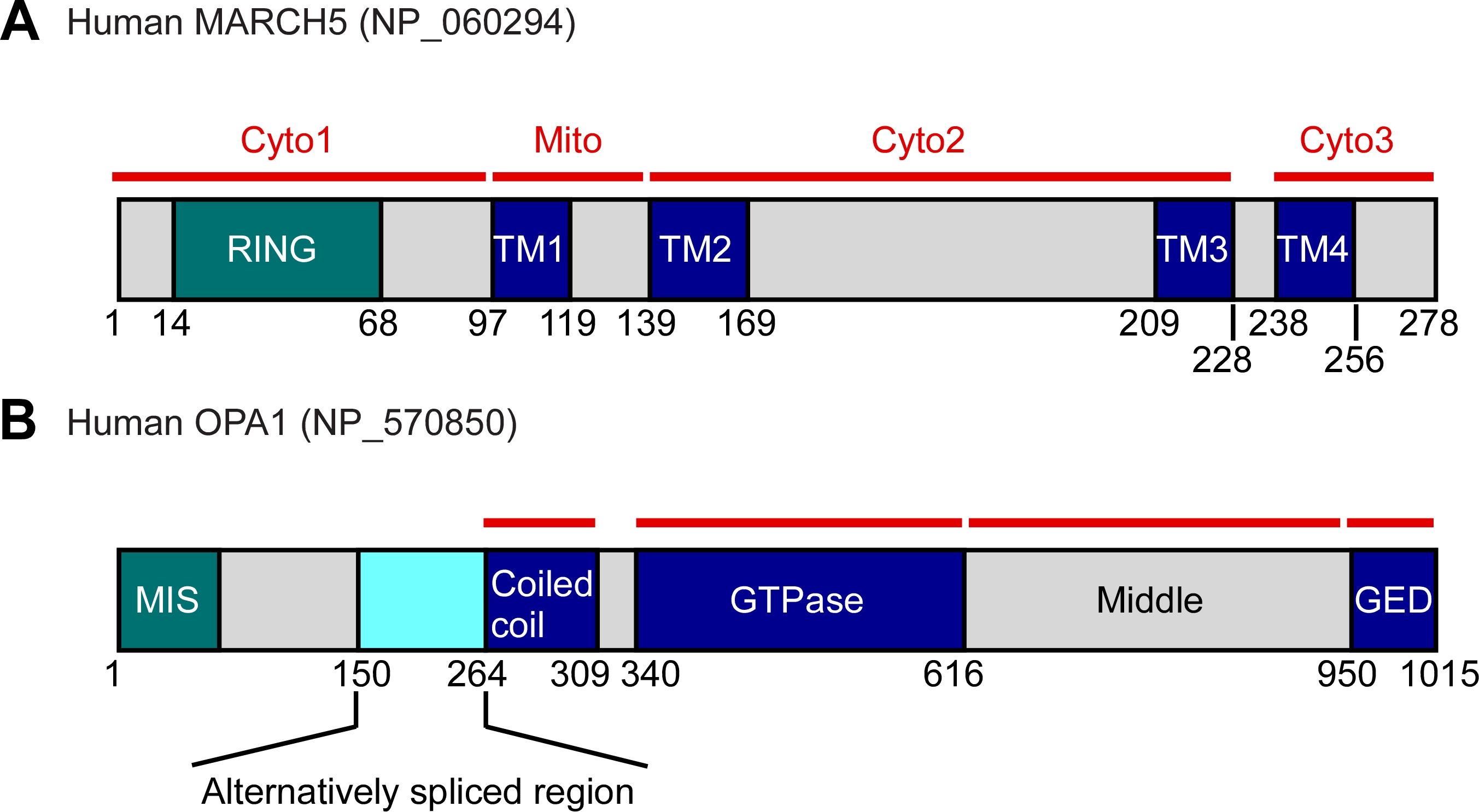


**Supplementary Figure 6. Structural domains of MARCH5 (NP_060294) (A) and OPA1 (NP_570850) (B) in human.** **(A)** MARCH5 sequence contains a RING domain at the N terminus and four TM domains . Four regions in red lines were fetched and used for the estimation of dN and dS substitution rate variation of MARCH5 at the domain level. ‘Cyto1’ indicates the cytosolic region 1 and ‘Mito’ indicates the mitochondrial region. **(B)** OPA1 contains the first coiled-coil domain, a GTPase domain (i.e., Dynamin domain PF00350), a middle domain and a GTPase effector domain (GED) that contains the second coiled-coil domain . The four domain regions were used in the domain-level analysis of substitution rate variation of OPA1. The first coiled-coil region is involved in protein-protein interactions. A mitochondrial import sequence (MIS) followed by an alternatively spliced region are on the upstream of the first coiled-coil domain.


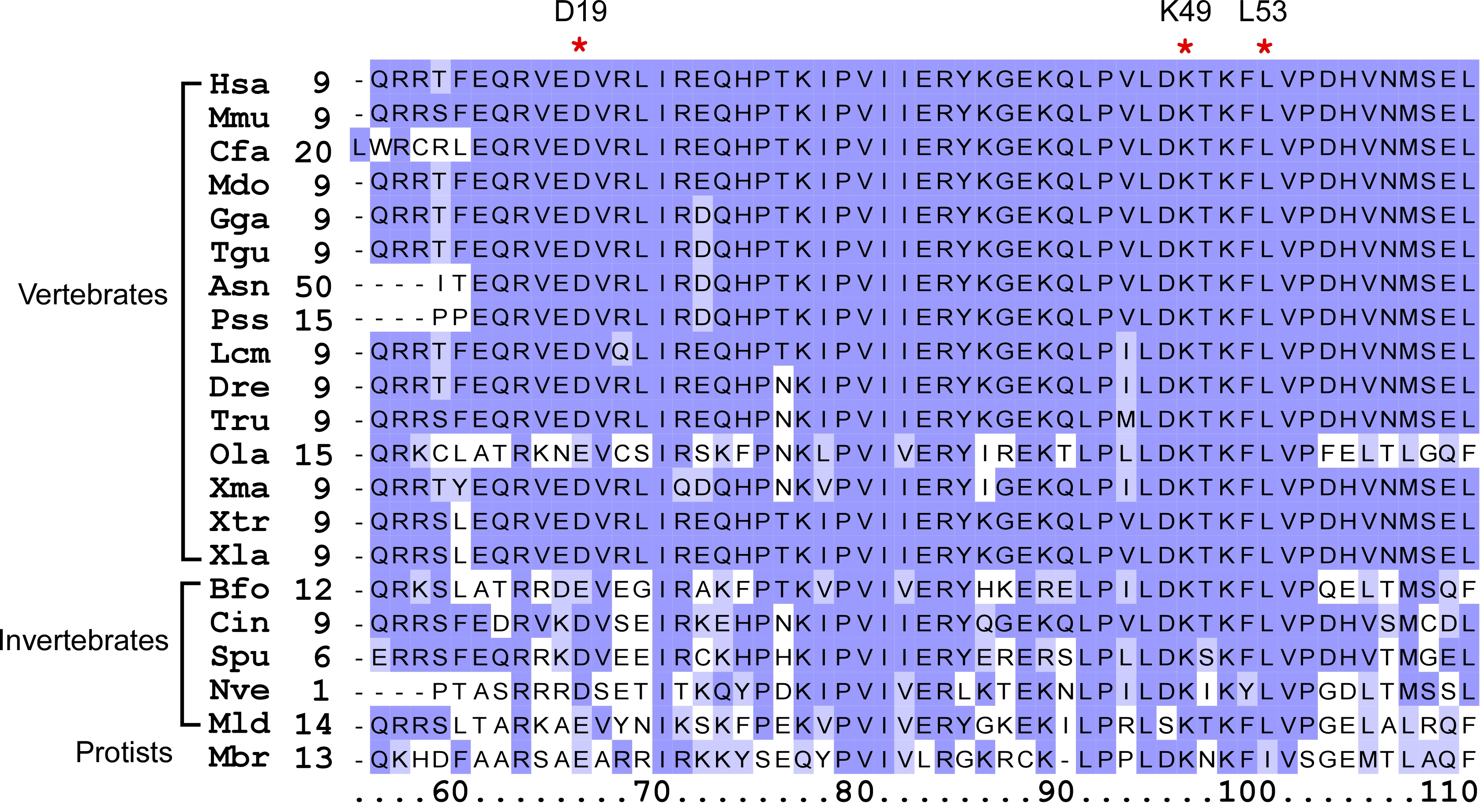


**Supplementary Figure 7. Alignment of LC3B in representative metazoan species and *M. brevicollis*.** Compared to LC3A and GABARAP proteins, LC3B appears to have the highest affinity with FUNDC1 . Red asterisks indicate the LC3B amino acid residues D19, K49 and L53, which respectively associate with Y18, S17 and V20 of FUNDC1 in human. Ola indicates *Oryzias latipes* and Tru indicates *Takifugu rubripes*. The information on other species is listed in Table 1.

**Supplementary Table 2.** Chromosome localizations of FUNDC1 and FUNDC2 in four vertebrate species, namely, human *Homo Sapiens* (Hsa), pig *Sus scrofa* (Ssc), chicken *Gallus gallus* (Gga) and zebrafish *Danio rerio* (Dre).

| Lineage | Paralogs | RefSeq ID | Ensembl Gene ID | Chr. | Start-End:Strand |
| --- | --- | --- | --- | --- | --- |
| Mammals | Hsa_FUNDC1 | NP_776155.1 | ENSG00000069509 | X | 44523639-44543001:-1 |
| Mammals | Hsa_FUNDC2 | NP_076423.2 | ENSG00000165775 | X | 155025980-155060303:1 |
| Mammals | Ssc_FUNDC1 | XP_003135086.1 | ENSSSCG00000012258 | X | 44109888-44121368:-1 |
| Mammals | Ssc_FUNDC2 | NP_998908.1 | ENSSSCG00000012817 | X | 143115683-143129036:1 |
| Birds | Gga_FUNDC1 | NP_001263292.1 | ENSGALG00000028187 | 1 | 111018604-111035154:1 |
| Birds | Gga_FUNDC2 | XP_420194.1 | ENSGALG00000029585 | 4 | 2184257-2188075:1 |
| Fishes | Dre_FUNDC1 | NP_001002711.2 | ENSDARG00000040822 | 9 | 33974364-33976015:-1 |
| Fishes | Dre_FUNDC2 | NP_001005954.1 | ENSDARG00000103740 | 21 | 44563677-44570464:-1 |

NOTE.—Obtained from Ensembl release 86.

**Supplementary Table 3.** Likelihood ratio tests (LRT) for asymmetrical sequence evolution in FUNDC1 and FUNDC2 by using two-ratio models compared with the one-ratio model.

| Foreground lineage | 2×ΔLn *L* (One ratio vs. Two ratios)*^a^* | *P-*value | Estimates of parameters^b^ |
| --- | --- | --- | --- |
| Vertebrate FUNDC1 | 26.023 | 3.37E-07 | ω_B_ = 0.104, ω_F_ = 0.047 |
| Vertebrate FUNDC2 | 26.023 | 3.37E-07 | ω_B_ = 0.047, ω_F_ = 0.104 |

^a^ Degrees of freedom (df) = 1 and ΔLn *L* = ln *L*_1_ – ln *L*_0_, where ln *L*_1_ was the likelihood value of two-ratio model and ln *L*_0_ was the likelihood value of one-ratio model M0.

^b^ ω_F_ means the ω ratio for the foreground lineage and ω_B_ means the ω ratio for the background lineage. The ω value of one-ratio model was 0.074.

**Supplementary Table 4.** Estimates of the coefficient of functional divergence type I analysis in the clades of FUNDC1 and FUNDC2 in vertebrates.

|  | FUNDC1/FUNDC2 | Bootstrapped FUNDC1/FUNDC2 |
| --- | --- | --- |
| ThetaML | 0.539 | 0.556 |
| AlphaML | 0.776 | 0.760 |
| SE Theta | 0.156 | 0.157 |
| LRT Theta | 11.978 | 13.969 |
| *P*-value | 5.38E-04 | 1.86E-04 |

**Supplementary Table 5. Protein-protein interactions of human FUNDC2 evidenced by high-throughput experiments.**

| Gene | Interaction evidence^a^ | Protein (UniProt ID)^b^ | Function^b^ |
| --- | --- | --- | --- |
| SEC62 | Huttlin (Affinity Capture-MS) | Translocation protein SEC62 (Q99442) | Acts as component of the Sec62/63 complex which is involved in SRP-independent post-translational translocation across the endoplasmic reticulum and functions together with the Sec61 complex and KAR2 in a channel-forming translocon complex. |
| FAF2 | Huttlin (Affinity Capture-MS) | FAS-associated factor 2 (Q96CS3) | Plays an important role in endoplasmic reticulum-associated degradation (ERAD) that mediates ubiquitin-dependent degradation of misfolded endoplasmic reticulum proteins. |
| FUNDC1 | Huttlin (Affinity Capture-MS) | FUN14 domain-containing protein 1 (Q8IVP5) | Acts as an activator of hypoxia-induced mitophagy, an important mechanism for mitochondrial quality control. |
| DHCR24 | Huttlin (Affinity Capture-MS) | Delta(24)-sterol reductase (Q15392) | Catalyzes the reduction of the delta-24 double bond of sterol intermediates. Protects cells from oxidative stress by reducing caspase 3 activity during apoptosis induced by oxidative stress. Also protects against amyloid-beta peptide-induced apoptosis. |
| TP53I3 | Stelzl (Two-hybrid) | Quinone oxidoreductase PIG3 (Q53FA7) | May be involved in the generation of reactive oxygen species (ROS). |
| USP13 | Stelzl (Two-hybrid) | Ubiquitin carboxyl-terminal hydrolase 13 (Q92995) | Deubiquitinase that mediates deubiquitination of target proteins such as BECN1, MITF, SKP2 and USP10 and is involved in various processes such as autophagy and ERAD. |
| NACA | Stelzl (Two-hybrid) | Nascent polypeptide-associated complex subunit alpha (Q13765) | Prevents inappropriate targeting of non-secretory polypeptides to the endoplasmic reticulum. |
| NSF | Stelzl (Two-hybrid) | Vesicle-fusing ATPase (P46459) | Catalyzes the fusion of transport vesicles within the Golgi cisternae. Is also required for transport from the endoplasmic reticulum to the Golgi stack. |
| SHMT2 | Castello (Affinity Capture-RNA) | Serine hydroxymethyltransferase, mitochondrial (P34897) | Contributes to the de novo mitochondrial thymidylate biosynthesis pathway via its role in glycine and tetrahydrofolate metabolism. |
| GOLT1B | Hein (Affinity Capture-MS) | Vesicle transport protein GOT1B (Q9Y3E0) | May be involved in fusion of endoplasmic reticulum-derived transport vesicles with the Golgi complex. |

^a^ Literatures: Huttlin (Affinity Capture-MS) , Stelzl (Two-hybrid) , Castello (Affinity Capture-RNA) and Hein (Affinity Capture-MS) .

^b^ Protein information is from UniProt (<http://www.uniprot.org/>).

**Supplementary Table 6. LRT for asymmetrical sequence evolution in BNIP3 and NIX in vertebrates by using two-ratio models compared with the one-ratio model.**

| Foreground lineage | 2×ΔLn *L* (One ratio vs. Two ratios)*^a^* | *P-*value | Estimates of parameters^b^ |
| --- | --- | --- | --- |
| Vertebrate BNIP3 | 9.51 | 2.05E-03 | ω_B_ = 0.095, ω_F_ = 0.068 |
| Vertebrate NIX | 9.51 | 2.05E-03 | ω_B_ = 0.068, ω_F_ = 0.095 |

^a^ df = 1 and ΔLn *L* = ln *L*_1_ – ln *L*_0_, where ln *L*_1_ was the likelihood value of two-ratio model and ln *L*_0_ was the likelihood value of one-ratio model M0.

^b^ ω_F_ means the ω ratio for the foreground lineage and ω_B_ means the ω ratio for the background lineage. The ω value of one-ratio model was 0.081.

**Supplementary Table 7.** LRT for divergence in selective pressure among BNIP3 and NIX in vertebrates by using clade model C.

| 2×ΔLn *L* (M2a_rel vs. Clade Model C)*^a^* | *P-*value | Site class | p^b^ | ωClade_BNIP3^c^ | ωClade_NIX^c^ |
| --- | --- | --- | --- | --- | --- |
| 1.579 | 0.21 | 0 | 0.53 | 0.020 | 0.020 |
|  |  | 1 | 0.06 | 1 | 1 |
|  |  | 2 | 0.40 | 0.156 | 0.185 |

^a^ df = 2 and ΔLn *L* = Ln *L*1 – Ln *L*0, where Ln *L*1 was the likelihood value of clade model C and Ln *L*0 (-10104.238) was the likelihood value of null model M2a_rel.

^b^ Proportion of sites evolving under different site classes.

^c^ Measure of natural selection acting on the BNIP3 clade (ωClade_BNIP3) or NIX clade (ωClade_NIX).

**Supplementary Table 8.** LRT for detecting variable selection pressures among different lineages in animals, by using two-ratio models compared with the one-ratio model.

| Gene | Foreground lineage^a^ | Estimates of parameters^b^ | 2×ΔLn *L*^c^ | Adjusted *P*-value^d^ |
| --- | --- | --- | --- | --- |
| FUNDC1 | Teleost fishes | ω_B_ =0.079; ω_F_ = 0.033 | 13.60 | **3.4E-04** |
|  | Mammals | ω_B_ =0.062; ω_F_ = 0.073 | 0.69 | 0.407 |
|  | Land vertebrates | ω_B_ =0.033; ω_F_ = 0.079 | 13.60 | **3.4E-04** |
| MARCH5 | Teleost fishes | ω_B_ =0.029; ω_F_ = 0.015 | 6.50 | **0.016** |
|  | Mammals | ω_B_ =0.024; ω_F_ = 0.028 | 0.26 | 0.609 |
|  | Land vertebrates | ω_B_ =0.015; ω_F_ = 0.029 | 6.50 | **0.016** |
| BNIP3 | Teleost fishes | ω_B_ =0.056; ω_F_ = 0.085 | 5.84 | **0.016** |
|  | Mammals | ω_B_ =0.074; ω_F_ = 0.046 | 11.30 | **0.002** |
|  | Land vertebrates | ω_B_ =0.085; ω_F_ = 0.056 | 5.84 | **0.016** |
| NIX | Teleost fishes | ω_B_ =0.076; ω_F_ = 0.103 | 6.03 | **0.014** |
|  | Mammals | ω_B_ =0.105; ω_F_ = 0.058 | 20.15 | **2.1E-05** |
|  | Land vertebrates | ω_B_ =0.103; ω_F_ = 0.076 | 6.03 | **0.014** |

^a^ The relationship between teleost fishes and land vertebrates is displayed in Supplementary Figure 2.

^b^ ω_F_ was the ω ratio for the foreground lineage and ω_B_ was the ω ratio for the background lineage.

^c^ ΔLn *L* = ln *L*1 – ln *L*0, where ln *L*1 was the likelihood value of two-ratio model and ln *L*0 was the likelihood value of one-ratio model.

^d^ For each gene, the differences between ω_F_ and ω_B_ in different lineages were corrected using Benjamini-Hochberg multiple correction and are shown in bold at the significant level (*P*-value < 0.05).

**Supplementary Table 9.** Estimation of dN and dS substitution rate variation across the entire gene sequences of the proteins in the signaling pathway of hypoxia-induced mitophagy in the lineages of teleost fishes, mammals and land vertebrates.

| Gene | Lineage | dN | dS | ω (dN/dS) |
| --- | --- | --- | --- | --- |
| HIF-1α | Teleost fishes | 0.239 | 1.639 | **0.156** |
|  | Mammals | 0.035 | 0.409 | 0.084 |
|  | Land vertebrates | 0.073 | 0.882 | 0.081 |
| SRC | Teleost fishes | 0.076 | 1.896 | **0.039** |
|  | Mammals | 0.034 | 0.802 | 0.025 |
|  | Land vertebrates | 0.029 | 0.507 | 0.022 |
| DRP1 | Teleost fishes | 0.066 | 1.666 | **0.037** |
|  | Mammals | 0.038 | 0.818 | 0.030 |
|  | Land vertebrates | 0.018 | 0.404 | 0.022 |
| MFN1 | Teleost fishes | 0.229 | 1.329 | **0.162** |
|  | Mammals | 0.067 | 1.106 | 0.067 |
|  | Land vertebrates | 0.037 | 0.423 | 0.087 |
| MFN2 | Teleost fishes | 0.055 | 1.561 | **0.036** |
|  | Mammals | 0.026 | 1.206 | 0.023 |
|  | Land vertebrates | 0.016 | 0.481 | 0.029 |
| OPA1 | Teleost fishes | 0.057 | 1.693 | 0.036 |
|  | Mammals | 0.044 | 1.304 | 0.035 |
|  | Land vertebrates | 0.018 | 0.546 | 0.035 |

^a^ dN means the number of synonymous substitutions per synonymous site. dS means the number of nonsynonymous substitutions per nonsynonymous site. dN and dS were estimated from the SNAP program (see Materials and Methods). The ω value in teleost fishes was compared with those in mammals and land vertebrates, and the higher ω value is in bold.

**Supplementary Table 10.** Estimation of substitution rate variation in individual domains of OPA1 in the lineages of teleost fishes, mammals and land vertebrates.

| Gene | Domain^a^ | Teleost fishes^b^ | Mammals^b^ | Land vertebrates^b^ |
| --- | --- | --- | --- | --- |
| OPA1 | Coiled coil (264-309 aa) | 0.009 | **0.017** | **0.017** |
|  | GTPase (340-616 aa) | **0.018** | 0.007 | 0.010 |
|  | Middle (617-949 aa) | **0.018** | 0.008 | 0.010 |
|  | GED (950-1015 aa) | **0.016** | 0.012 | 0.013 |

^a^ Domain regions of OPA1 are displayed in Supplementary Figure 6B.

^b^ The ω value (=dN/dS) in teleost fishes was compared with those in mammals and land vertebrates, and the higher ω value is in bold. dN and dS were estimated from the SNAP program.

**Supplementary Note 1. Molecular evolutionary analyses of vertebrate FUNDC1 and FUNDC2 after gene duplication**

Gene duplication has long been thought as a primary source of material for the origin of evolutionary novelties, including new gene functions and expression patterns . Changes in protein function may then yield different evolutionary constraints on gene copies after duplications . To assess to what extent natural selection has been exerted on the FUN14 domain-containing proteins during vertebrate evolution and how the selective pressure is different among the gene copies after gene duplication event, molecular evolutionary analysis was carried out at the coding region level.

The nonsynonymous-to-synonymous ratio (ω = dN/dS) provides a measure of the selection pressure to which a gene pair is subject. Values of ω < 1, ω = 1, and ω > 1 indicate negative purifying selection, neutral evolution, and positive selection, respectively. Different codon substitution-based evolutionary models in PAML were used to estimate ω in subfamilies of FUNDC1 and FUNDC2 (see Materials and Methods). One ratio model, which assumes all lineages to have the same evolutionary rate, estimated the mean ω value was 0.074. The low ω ratio reflected strong purifying selection during most of the evolution of FUN14 domain-containing proteins. The LRTs for the comparisons of two-ratio branch models, which assigned different ω ratios for individual subfamilies, with the one-ratio model indicated asymmetrical evolution after gene duplication for the subfamilies of FUNDC1 and FUNDC2 (*P*-value = 3.37E-07 in Supplementary Table 3). Furthermore, to test whether FUNDC1 and FUNDC2 underwent divergent selection pressures in a subregion of the coding sequence following gene duplication event, the clade model C, which allows heterogeneous ω values across both sequences and branches leading to each paralogous subfamily, was applied. The LRT for the comparison of clade model C with site-specific null model M2a_ref, presented significant divergent selection pressures at sequence level in vertebrate FUNDC1 and FUNDC2 (*P*-value = 2.5E-09 in Table 2). More than half of sites (57%) evolved under very strong purifying selection (ω = 0.018), while the other sites (43%) evolved under divergent selective pressures, with strong purifying selection in FUNDC1 clade (ωClade_FUNDC1 = 0.089 in Table 2) and weaker purifying selection in FUNDC2 clade (ωClade_FUNDC2 = 0.248). Finally, branch-site model A for the foreground clade of FUNDC2 detected relaxed selective constraints probably exerted somewhere along the FUNDC2 sequences. Thus, the higher estimates of ω ratio for sites evolving under divergent selection pressures in the FUNDC2 subfamily was explained by relaxed selective pressure instead of increased positive Darwinian selection at some sites.

We next examined the phylogeny-based functional divergence of the FUN14 domain-containing protein family after duplication using type I and type II divergence analyses (see Materials and Methods). Type I divergence results in altered evolutionary rates at certain amino acid residues while type II produces conserved residues in two clades but radical shifts of physicochemical properties . Significant functional divergence type I was obtained in the lineages leading to vertebrate FUNDC1 and FUDNC2 (LRT Theta = 14 and *P*-value = 1.86E-04 in Supplementary Table 4), revealing that the two paralogous subfamilies experienced distinct functional constraints during their independent evolution after gene duplication. No significant functional divergence type II was found. Three sites likely to be responsible for functional divergence type I were also identified (see Materials and Methods, labeled with green dots in Figure 1B). Amino acid residues at these sites are highly conserved in sequences from one subfamily but variable in the other, representing a shift in their evolutionary constraints between the two subfamilies, and they may lead to a change on their functional roles. One functionally divergent site, Q112 referenced on the human FUNDC1 sequence, is in the cytosol-exposed loop between TM2 and TM3. Two other sites, E19 and E25, are within or next to the LIR motif (Figure 1B), consistent with the observation by Carretero-Paulet et al. that predicted functionally divergent amino acids may not be functionally essential, but some of these residues are proximal to important functional residues . Obviously, functional roles of these amino acids identified to be involved in putative functional shifts during the FUNDC1 and FUNDC2 evolution in vertebrates need to be experimentally determined.

**Supplementary References**

Belenguer, P., and Pellegrini, L. (2013). The dynamin GTPase OPA1: more than mitochondria? *Biochim. Biophys. Acta* 1833**,** 176-183. doi: 10.1016/j.bbamcr.2012.08.004

Braasch, I., Gehrke, A. R., Smith, J. J., Kawasaki, K., Manousaki, T., Pasquier, J., et al. (2016). The spotted gar genome illuminates vertebrate evolution and facilitates human-teleost comparisons. *Nat. Genet.* 48**,** 427-437. doi: 10.1038/ng.3526

Carretero-Paulet, L., Albert, V. A., and Fares, M. A. (2013). Molecular evolutionary mechanisms driving functional diversification of the HSP90A family of heat shock proteins in eukaryotes. *Mol. Biol. Evol.* 30**,** 2035-2043. doi: 10.1093/molbev/mst113

Castello, A., Fischer, B., Eichelbaum, K., Horos, R., Beckmann, B. M., Strein, C., et al. (2012). Insights into RNA biology from an atlas of mammalian mRNA-binding proteins. *Cell* 149**,** 1393-1406. doi: 10.1016/j.cell.2012.04.031

Chen, Z., Liu, L., Cheng, Q., Li, Y., Wu, H., Zhang, W., et al. (2017). Mitochondrial E3 ligase MARCH5 regulates FUNDC1 to fine-tune hypoxic mitophagy. *EMBO Rep.* 18**,** 495-509. doi: 10.15252/embr.201643309

Edgar, R. C. (2004). MUSCLE: multiple sequence alignment with high accuracy and high throughput. *Nucleic Acids Res.* 32**,** 1792-1797. doi: 10.1093/nar/gkh340

Gu, X. (1999). Statistical methods for testing functional divergence after gene duplication. *Mol. Biol. Evol.* 16**,** 1664-1674.

Gu, X. (2006). A simple statistical method for estimating type-II (cluster-specific) functional divergence of protein sequences. *Mol. Biol. Evol.* 23**,** 1937-1945. doi: 10.1093/molbev/msl056

Hein, M. Y., Hubner, N. C., Poser, I., Cox, J., Nagaraj, N., Toyoda, Y., et al. (2015). A human interactome in three quantitative dimensions organized by stoichiometries and abundances. *Cell* 163**,** 712-723. doi: 10.1016/j.cell.2015.09.053

Hong, Z., Bednarek, S. Y., Blumwald, E., Hwang, I., Jurgens, G., Menzel, D., et al. (2003). A unified nomenclature for Arabidopsis dynamin-related large GTPases based on homology and possible functions. *Plant Mol. Biol.* 53**,** 261-265.

Huttlin, E. L., Ting, L., Bruckner, R. J., Gebreab, F., Gygi, M. P., Szpyt, J., et al. (2015). The BioPlex Network: A Systematic Exploration of the Human Interactome. *Cell* 162**,** 425-440. doi: 10.1016/j.cell.2015.06.043

Liu, L., Feng, D., Chen, G., Chen, M., Zheng, Q., Song, P., et al. (2012). Mitochondrial outer-membrane protein FUNDC1 mediates hypoxia-induced mitophagy in mammalian cells. *Nat. Cell Biol.* 14**,** 177-185. doi: 10.1038/ncb2422

Meyer, A., and Van de Peer, Y. (2005). From 2R to 3R: evidence for a fish-specific genome duplication (FSGD). *Bioessays* 27**,** 937-945. doi: 10.1002/bies.20293

Ohno, S. (1970). *Evolution by gene duplication.* Berlin: Springer-Verlag.

Pyron, R. A. (2011). Divergence time estimation using fossils as terminal taxa and the origins of Lissamphibia. *Syst. Biol.* 60**,** 466-481. doi: 10.1093/sysbio/syr047

Stelzl, U., Worm, U., Lalowski, M., Haenig, C., Brembeck, F. H., Goehler, H., et al. (2005). A human protein-protein interaction network: a resource for annotating the proteome. *Cell* 122**,** 957-968.

Taylor, C. T., and McElwain, J. C. (2010). Ancient atmospheres and the evolution of oxygen sensing via the hypoxia-inducible factor in metazoans. *Physiology* 25**,** 272-279. doi: 10.1152/physiol.00029.2010

Taylor, J. S., and Raes, J. (2004). Duplication and divergence: the evolution of new genes and old ideas. *Annu. Rev. Genet.* 38**,** 615-643. doi: 10.1146/annurev.genet.38.072902.092831

UniProt, C. (2015). UniProt: a hub for protein information. *Nucleic Acids Res.* 43**,** D204-212. doi: 10.1093/nar/gku989

Yang, Z. (1997). PAML: a program package for phylogenetic analysis by maximum likelihood. *Comput. Appl. Biosci.* 13**,** 555-556.
